# Pro-Oncogenic c-Met/EGFR, Biomarker Signatures of the Tumor Microenvironment are Clinical and Therapy Response Prognosticators in Colorectal Cancer, and Therapeutic Targets of 3-Phenyl-2H-benzo[e][1,3]-Oxazine-2,4(3H)-Dione Derivatives

**DOI:** 10.3389/fphar.2021.691234

**Published:** 2021-08-27

**Authors:** Bashir Lawal, Yu-Chi Wang, Alexander T. H. Wu, Hsu-Shan Huang

**Affiliations:** ^1^ PhD Program for Cancer Molecular Biology and Drug Discovery, College of Medical Science and Technology, Taipei Medical University and Academia Sinica, Taipei, Taiwan; ^2^ Graduate Institute for Cancer Biology and Drug Discovery, College of Medical Science and Technology, Taipei Medical University, Taipei, Taiwan; ^3^ Department of Obstetrics and Gynecology, Tri-Service General Hospital, National Defense Medical Center, Taipei, Taiwan; ^4^ TMU Research Center of Cancer Translational Medicine, Taipei Medical University, Taipei, Taiwan; ^5^ The PhD Program of Translational Medicine, College of Science and Technology, Taipei Medical University, Taipei, Taiwan; ^6^ Clinical Research Center, Taipei Medical University Hospital, Taipei Medical University, Taipei, Taiwan; ^7^ Graduate Institute of Medical Sciences, National Defense Medical Center, Taipei, Taiwan; ^8^ School of Pharmacy, National Defense Medical Center, Taipei, Taiwan; ^9^ PhD Program in Drug Discovery and Development Industry, College of Pharmacy, Taipei Medical University, Taipei, Taiwan

**Keywords:** colorectal cancer, genetic and epigenetic alterations, cancer-associated fibroblast, immune infiltration, small molecule, NSC777205, NSC777207

## Abstract

Genetic and environmental factors play important roles in cancer progression, metastasis, and drug resistance. Herein, we used a multiomics data analysis to evaluate the predictive and prognostic roles of genetic and epigenetic modulation of c-MET (hepatocyte growth factor receptor)/epidermal growth factor receptor (EGFR) in colorectal cancer (CRC). First, we found that overexpressions of c-MET/EGFR were associated with the infiltration of tumor immune cells and cancer-associated fibroblasts, and were of prognostic relevance in CRC cohorts. We also observed that genetic alterations of c-MET/EGFR in CRC co-occurred with other gene alterations and were associated with overexpression of messenger (m)RNA of some cancer hallmark proteins. More specifically, DNA-methylation and somatic copy number alterations of c-MET/EGFR were associated with immune infiltration, dysfunctional T-cell phenotypes, and poor prognoses of the cohorts. Moreover, we describe two novel gefitinib-inspired small molecules derivatives of 3-phenyl-2H-benzo[e] [1,3]-oxazine-2,4(3H)-dione, NSC777205 and NSC777207, which exhibited wide-spectrum antiproliferative activities and selective cytotoxic preference for drug-sensitive and multidrug-resistant melanoma, renal, central nervous system, colon, and non-small cell lung cancer cell lines. We further provided in silico mechanistic evidence implicating c-MET/EGFR/phosphatidylinositol 3-kinase (PI3K)-mammalian target of rapamycin (mTOR) inhibition in anticancer activities of those compounds. Our overall structure-activity relationship study revealed that the addition of an –OCH_3_ group to salicylic core of NSC777207 was not favorable, as the added moiety led to overall less-favorable drug properties as well as weaker anticancer activities compared to the properties and activities demonstrated by NSC777205 that has no –OCH_3_ substituent group. Further *in vitro* and *in vivo* analyses in tumor-bearing mice are ongoing in our lab to support this claim and to unravel the full therapeutic efficacies of NSC777205 and NSC777207 in CRC.

## Introduction

According to 2020 global cancer statistics, with more than 1.9 million new cases and 935,000 deaths, colorectal cancer (CRC) ranked third in terms of incidence, but second in terms of mortality in 2020 (Sung et al., 2021). CRC is regarded as a marker of socioeconomic development, as incidence rates tend to rise uniformly with an increasing human development index (HDI) in countries undergoing major transitions (Bray, 2014; Fidler et al., 2016). In particular, incidence rates have been steadily increasing in Asia, Europe, and South America (Arnold et al., 2017; Arnold et al., 2020). These increases correspond to adoption of lifestyles that are associated with risk factors, such as being overweight, having decreased physical activity, and becoming more sedentary (Siegel et al., 2020). In addition, increased consumption of cigarettes, alcohol, and red or processed meats, and decreased intake of dairy products, grains, and fibers in those regions have greatly contributed to the observed trends (Clinton et al., 2020).

Both genetic and environmental factors play important roles in the etiology of CRC. To date, several molecular hallmarks have been associated with CRC (Colussi et al., 2013). These hallmarks of CRC are acquired through progressive accumulation of genetic and epigenetic alterations that inactivate tumor-suppressor genes and activate oncogenes (Grady and Carethers, 2008; Kuipers et al., 2015). At the molecular level, activation of growth factor receptors (GFRs) was shown to be involved in the rapid growth of cancer cells (Normanno et al., 2006). The etiology of most cancers can be linked to aberrant intra- and intercellular communication associated with GFR-mediated pathways. Activated GFRs aid blood vessel formation, cell migration, metastasis, and the inhibition of apoptosis. Among these GFRs, the epidermal growth factor receptor (EGFR) and hepatocyte growth factor receptor (HGFR) play central roles in the pathogenesis and progression of different carcinoma types (Normanno et al., 2001).

HGFR, also known as tyrosine-protein kinase (c-Met), is a tyrosine kinase receptor (RTK) that in humans is encoded by the *MET* gene, while the EGFR belongs to the ErbB family of RTKs, and is a trans-membrane protein. The binding of ligands to the extracellular domain of these receptors induces the formation of receptor homo- or heterodimers and subsequent activation of the intrinsic tyrosine kinase domain (Oprita et al., 2021), which facilitates recruitment of proteins that initiate a signaling cascade, integrating numerous signaling pathways that lead to specific cellular responses that favor angiogenesis, high nutrient supplies, cell migration, tumor growth, and metastasis of CRC (Sattler et al., 2011; An et al., 2018).

Several treatment modalities exist, including immunotherapy, radiotherapy, neoadjuvant and palliative chemotherapies, laparoscopic surgery for primary disease, and more-aggressive resection of metastatic disease, and these provide alternatives for patients with primary and metastatic CRC (Stintzing, 2014; Kuipers et al., 2015). However, these treatment strategies have limited success rates in terms of prognoses and long-term survival. For these reasons, searching for relevant predictive biomarkers that can inform treatment decisions and developing novel therapeutic strategies with high efficacy and minimal side effects are impetuses for the research world. To this end, we used a multiomics data analysis to evaluate the predictive and prognostic roles of genetic and epigenetic modulation of c-MET/EGFR in CRC. Moreover, we describe two novel gefitinib-inspired small molecules, NSC777205 and NSC777207, with wide-spectrum antiproliferative activities and selective cytotoxic preferences for melanoma, renal, central nervous system (CNS), colon, and non-small cell lung cancer (NSCLC) cell lines, and provide *in silico* mechanistic evidence implicating c-MET/EGFR/phosphatidylinositol 3-kinase (PI3K)/mammalian target of rapamycin (mTOR) inhibition in anticancer activities of these compounds.

## Materials and Methods

### *In Silico* Evaluation of the Drug Likeness, Pharmacokinetics, Acute Toxicity, and Cytotoxic Activities of NSC777205 and NSC777207 Against Cancer Cell Lines

We analyzed the drug-likeness, PKs, medicinal chemistry, and toxicity of NSC777205 and NSC777207 using SwissADME software (http://www.swissadme.ch) (Daina et al., 2017), and computer-aided Prediction of Biological Activity Spectra (PASS) web resources (http://way2drug.com/dr) (Poroikov et al., 2019). We used the blood-brain barrier (BBB) Prediction Server (https://www.cbligand.org/BBB/) which operates based on support vector machine (SVM) and LiCABEDS algorithms on four types of fingerprints of 1,593 reported compounds (Liu et al., 2014) to analyze the BBB-permeation ability of NSC777205 and NSC777207 based on the permeation threshold of 0.02. In addition, we also used the Brain Or IntestinaL EstimateD permeation method (BOILED-Egg) model (Daina and Zoete, 2016) to further analyze the brain- and intestinal-permeation abilities of the compounds based on their lipophilicity and polarity. We used the CLC-Pred (Cell Line Cytotoxicity Predictor) modules of the PASS server (http://www.way2drug.com/PASSonline) (Poroikov et al., 2019) created based on a training set of data on cytotoxicity retrieved from ChEMBLdb (vers. 23) (https://www.ebi.ac.uk/chembldb/), to predict the cytotoxic activities of NSC777205 and NSC777207 against various cancer cell lines.

### Differential Expression and Survival Analysis of Tyrosine-Protein Kinase/Epidermal Growth Factor Receptor in Colorectal Cancer

Expression levels of *c-MET/EGFR* in CRC tissues from The Cancer Genome Atlas (TCGA) database were compared with expression levels in normal tissues using the TNMplot module of the Kaplan-Meier Plotter (https://www.tnmplot.com/). Furthermore, we used the Human Protein Atlas (HPA) database (www.proteinatlas.org) to assess the immunohistochemical (IHC) profile of *c-MET/EGFR* in tumor samples from CRC patients. We used the MEXPRESS algorithm (https://mexpress.be/index.html) to depict the mechanisms of c-MET/EGFR dysregulation in colorectal cancer (Koch et al., 2019). To analyze the prognostic relevance of the gene signatures, we used the PREdiction of Clinical Outcomes from Genomic profiles (PRECOG) server (https://precog.stanford.edu/precog_metaZ.datatable.php) (Gentles et al., 2015) to split CRC patient cohorts into high and low *c-MET/EGFR* expression groups, by setting the median expression level as the expression threshold. Kaplan-Meier survival plots were used to present the survival ratio of cohorts with hazard ratios (HRs), 95% confidence intervals (CIs), and log-rank test *p* values.

### Analysis of the Effects of Tyrosine-Protein Kinase/Epidermal Growth Factor Receptor Expression, and Genetic and Epigenetic Alterations on Immune Infiltration, Dysfunctional T-Cell Phenotypes, and Prognostic Relevance in Colorectal Cancer

We used the cBioPortal server (http://www.cbioportal.org/) to mine the Colorectal Adenocarcinoma (TCGA, PanCancer Atlas) dataset for c-MET/EGFR genetic alterations including mutations, copy number alterations, gene mutation co-occurrences, and microbiome signatures and evaluated the prognostic relevance of the alterations in 594 CRC patients using survival analyses of the cohorts (Cerami et al., 2012; Gao et al., 2013). Protein-protein interaction (PPI) networks and functional enrichment analyses including Kyoto Encyclopedia of Genes and Genomes (KEGG) pathways and biological processes enriched in genes that were associated with c-MET/EGFR alterations were analyzed using the Search Tool for Retrieval of Interacting Genes (STRING, vers. 10.5, (https://www.string-db.org) with the adjusted threshold confidence set to 0.400 (Szklarczyk et al., 2019). We used the IMmune Estimation Resource (TIMER2.0) algorithm (http://timer.cistrome.org/) to analyze correlations of c-MET/EGFR expression with somatic copy number alterations and infiltration of six tumor-infiltrating immune cell subsets (B cells, cluster of differentiation 4 (CD4) T cells, CD8 T cells, macrophages, neutrophils, and dendritic cells) in CRC. We also used the TIMER server to analyze correlations of c-MET/EGFR expressions and cancer-associated fibroblast (CAF) infiltration. In addition, we analyzed the prognostic relevance of these associations by employing a protocol described in a previous study (Lawal et al., 2021a). Briefly, TCGA CRC cohorts on the TIMER server were categorized into four groups of low CAF+low c-MET/EGFR, low CAF+high c-MET/EGFR, high CAF+low c-MET/EGFR, and high CAF+high c-MET/EGFR and used Kaplan-Meier survival plots to analyze the cumulative survival of the cohorts. We also used Tumor Immune Dysfunction and Exclusion (TIDE) (http://tide.dfci.harvard.edu) (Jiang et al., 2018) to analyze the effects of genetic (somatic copy number) and epigenetic (DNA methylation) alterations of c-MET/EGFR on dysfunctional T-cell phenotypes, risk factors, and survival of CRC cohorts.

### *In Vitro* Anticancer Screening of NSC777205 and NSC777207

NSC777205 and NSC777207 were evaluated for *in vitro* anticancer activities against 60 panels of human tumor cancer cell lines representing leukemia (six cell lines), NSCLC (eight cell lines), colon cancer (seven cell lines), CNS cancers (six cell lines), melanomas (nine cell lines), ovarian cancer (seven cell lines), renal cancer (eight cell lines), prostate cancer (two cell lines), and breast cancer (five cell lines) through the National Cancer Institute (NCI). Ranges of 5,000–40,000 viable cancer cells were seeded into each well of 96-well plates and incubated at 37 °C with 5% CO_2_, 95% air, and 100% relative humidity for 24 h. After incubation, cells were treated with either NSC777205 or NSC777207 at a single dose of 10 μM and further incubated for 48 h (Shoemaker, 2006; Holbeck et al., 2010). A sulforhodamine B (SRB) staining protocol (Vichai and Kirtikara, 2006) was used to determine cell viability. After determining satisfactory antiproliferative activities of a single dose, both NSC777205, and NSC777207 were further subjected to multiple-dose screening at five concentrations of 0, 0.1, 1.0, 10, and 100 μM using the same protocol described above. The activity of each of the drug on each cell line was calculated using four measurements parameters as described here.

Growth inhibition (GI; %): [(Ti−Tz)/(C−Tz)] × 100 for concentrations for which Ti>/ = Tz or [(Ti−Tz)/Tz] x 100 for concentrations for which Ti < Tz.

50% GI (GI_50_) (μM) = [(Ti−Tz)/(C−Tz)] × 100 = 50.

Total growth inhibition (TGI; μM) = Ti = Tz.

50% Lethal concentration (LC_50_; μM) = [(Ti−Tz)/Tz] × 100 = −50.

In these equations, Tz is the absorbance at time 0; C is the absorbance of the control after 48 h without treatment; Ti is the absorbance of drug-treated cells after 48 h; GI_50_ is the concentration of the drug causing a 50% reduction in cell growth; TGI is the concentration causing complete inhibition of cell growth, and LC_50_ is the concentration causing 50% cell death. For a drug whose maximum dose tested (100 μM) did not meet the required effect on a particular cell line, the value for that parameter was expressed as greater than the maximum concentration tested (>100 μM).

### DTP-COMPARE Analysis and in Silico Identification of Mechanistic Targets for NSC777205 and NSC777207

We used the private COMPARE module of the NCI-COMPARE program (https://dtp.cancer.gov/databases_tools/compare.htm) to correlate anticancer activity fingerprints of NSC777205 and NSC777207 with NCI synthetic compounds, standard agents, and molecular targets based on established relationships between cell responses to therapy and gene expression profiles of the cell lines (Paull et al., 1989). The NSC numerical IDs were used as the “seed,” whereas GI_50_, TGI, and LC_50_ values were set as endpoints (Lawal et al., 2021b). Analyses were conducted based on program default settings of a minimum correlation of 0.1, minimum common cell line counts of 40, minimum coefficient of variation of 0.01, and maximum return of 200, while results were generated in rank ordered lists of the most highly correlated NCI compounds and targets. In addition to the COMPARE algorithm, we also identified potential targets of NSC777205 and NSC777207 using three different *in silico* target identification algorithms, including the PharmMapper Server, a pharmacophore mapping algorithm with statistical modules (http://lilab-ecust.cn/pharmmapper/index.html) (Liu et al., 2010), the SwissTargetPrediction algorithm (http://www.swisstargetprediction.ch/), which operates on the basis of similarity of the queried molecule with known active drugs (Gfeller et al., 2013), and the computer-aided PASS web resources (http://way2drug.com/dr) (Poroikov et al., 2019).

### Molecular Docking Study of NSC777205 and NSC777207 With Various Targets

Three-dimensional (3D) molecular ball-and-stick models in mol2 format of NSC777205 and NSC777207 were obtained using the Avogadro molecular builder and visualization tool vers. 1.XX (http://avogadro.cc/) (Hanwell et al., 2012), while 3D models of standard drugs, including dactolisib (CID: 11977753), gefitinib (CID: 123631), copanlisib (CID: 135565596), and crizotinib (CID: 11626560), were retrieved in sdf file format from the PubChem database (https://pubchem.ncbi.nlm.nih.gov/). All mol2 and sdf files were converted to pdb files using the PyMOL Molecular Graphics System, vers. 1.2r3pre (Schrödinger; https://pymol.org/edu/?q=educational/), while pdbqt files of ligands were generated from pdb files using AutoDock Vina (vers. 0.8, Scripps Research Institute, La Jolla, CA, United States) (Trott et al., 2010). Crystal structures of PI3K (PDB:3APC), (B) c-MET (PDB: 3RHK), (C) EGFR (PDB: 5EDP), and (D) mTOR (PDB: 5FLC) were obtained from the Protein Data Bank (https://www.rcsb.org/) in protein data bank (PDB) file format and subsequently converted into the Auto Dock Pdbqt format using AutoDock Vina tools. All receptors were charged, hydrogen atoms were added, and water (H_2_0) molecules were removed prior to docking (Lawal et al., 2021b). Docking experiments were performed with AutoDock Vina software using default settings, and at a docking exhaustiveness of 8, with all bonds in the ligand rotated freely while considering the receptor to be rigid. A grid box of 40 × 40 × 40 Å in the X, Y, and Z dimensions and a spacing of 1.0 Å were used (Lawal et al., 2021c). Docking outcomes were visualized in 2D conformations using the Discovery studio visualizer vers. 19.1.0.18287 (BIOVIA, San Diego, CA, United States) (Visualizer, 2020) and the protein-ligand interaction profiler (https://plip-tool.biotec.tu-dresden.de/plip-web/plip/index) (Salentin et al., 2015).

### Statistical Analysis

All statistical analyses were conducted according to each server's instructions. We used the Wilcoxon test to compare statistically significant differences in c-MET/EGFR expressions between CRC cohorts and healthy tissues. Purity adjustment and partial Spearman’s correlations were used to analyze c-MET/EGFR expression correlations with infiltration of CAFs and various immune cells. Gene alteration co-occurrences on the cbioportal were considered significant only at an adjusted *p* value of <0.05. The KEGG and gene ontologies (GOs) were visualized using GraphPad prism software. DTP-COMPARE analyses were conducted using Pearson’s correlations. All survival analyses are presented using Kaplan-Meier plots. GI was calculated relative to cells without drug treatment and the time-zero control. Statistical significance was denoted as *p < 0.05, **p < 0.01, and ***p < 0.001.

## Results

### Tyrosine-Protein Kinase/Epidermal Growth Factor Receptor are Deregulatory Expressed and are Associated With Oxaliplatin Resistance and Poor Prognoses of Colorectal Cancer Patients

**FIGURE 1:**
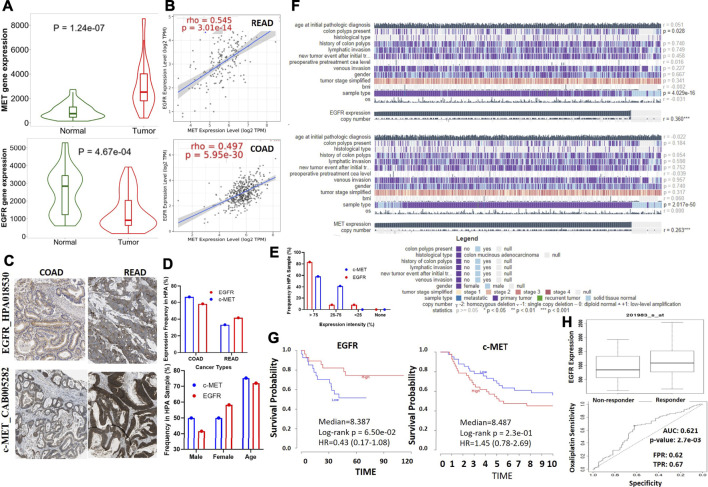
c-Met and epidermal growth factor receptor (EGFR) are overexpressed and are associated with poor prognoses of colorectal cancer patients. **(A)** Violine plots showing differential gene expression levels of c-Met/EGFR between colorectal cancer tumors and adjacent normal tissues. Green labels indicate normal tissues, and red labels indicate tumor samples. **(B)** Expression scatterplots of c-Met correlations with EGFR in COAD and READ. **(C)** Representative IHC staining of c-MET/EGFR protein expressions in colorectal cancer cohorts. **(D)** Frequencies, gender and age differences, and **(E)** expression intensities of EGFR/c-MET in COAD and READ samples. **(F)** Heat map summary of the mechanisms of c-Met/EGFR dysregulation in TCGA colorectal cancer cohorts. **(G)** Kaplan-Meier curve of the survival of colorectal cancer cohorts with high or low c-MET/EGFR expression levels. **(H)** Gene expression profile between oxaliplatin sensitive and resistance colorectal cancer cohorts. The strength of correlations between the genes is reflected by the purity-adjusted partial Spearman’s rho value and estimated statistical significance, where a value of *r* = 1 means a perfect positive correlation and a value of *r* = −1 means a perfect negative correlation. *p < 0.05; **p < 0.01; ***p < 0.001.

We conducted a differential expression analysis between the CRC tumorous and adjacent normal tissues in TCGA database. We found that c-MET expression is higher (*p* = 1.24e-07) while EGFR expression is lower (*p* = 4.67e-04) in colorectal cancer cohorts compared to adjacent normal cohorts**.** (Figure 1A). Correlation analyses also indicated that c-MET expression was strongly correlated with EGFR expressions in COAD (*r* = 0.497, *p* = 5.95e-30) and READ (*r* = 0.545, *p* = 3.01e-14) (Figure 1B). Furthermore, we explored the HPA database for IHC data of c-MET/EGFR protein expressions based on tissue microarrays (TMAs) in CRC cohorts (Figure 1C). We found that all CRC patient samples presented for EGFR (% 66.66 COAD and 33.33% READ) and c-MET (58.33% COAD and 41.66% READ) expression profiling were positive for c-MET (Antibody: CAB005282) and EGFR (Antibody: HPA018530) signals (Figure 1D). Mean ages of patients with high c-Met and EGFR expression profiles were 72.08 and 75.25 years, respectively. Totals of 58.33 and 83.33% of patients had very high intensities (>75%), while 41.66 and 8.33% had medium intensities of c-Met and EGFR expression, respectively (Figure 1E). Only 8.33% of patients had a low EGFR expression intensity (Figure 1E). Our analysis of the mechanisms of gene dysregulation indicated that EGFR expression is significantly associated with colon polyps (*p* = 0.028) and primary tumors *p* = 0.04029) but less associated with metastasis or recurrent tumor. Furthermore, both c-MET and EGFR expressions show significant correlation with copy number alterations in colorectal cancer (Figure 1F). To assess the prognostic relevance of gene signatures, we conducted a survival analysis and found that CRC cohorts with higher expression profiles of c-Met and cohorts with lower expression profiles of EGFR exhibited low survival rates (Figure 1G) and resistance to oxaliplatin chemotherapy.

### Tyrosine-Protein Kinase/Epidermal Growth Factor Receptor Expressions are Associated With Tumor Infiltration of Various Immune Cells and Cancer Associated Fibroblast, and Poor Prognoses of Colorectal Cancer Cohorts

**FIGURE 2:**
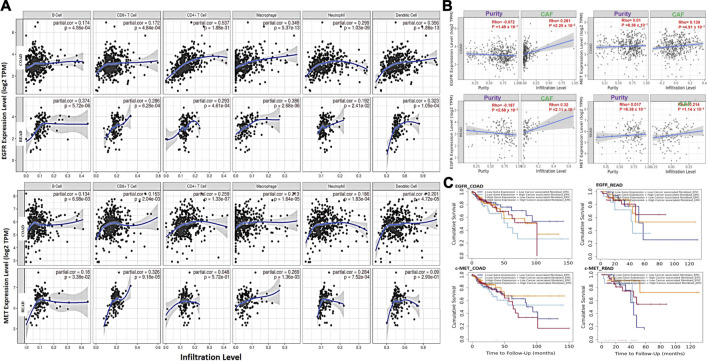
c-MET/epidermal growth factor receptor (EGFR) expressions are associated with tumor immune infiltration and poor prognoses of colorectal cancer cohorts. **(A)** Scatterplots showing correlations of c-MET/EGFR expressions with immune infiltration levels in colon adenocarcinoma (COAD) and rectum adenocarcinoma (READ). **(B)** Scatterplot showing purity-adjusted correlations of c-MET/EGFR expressions and CAF infiltration colon adenocarcinoma (COAD) and rectal adenocarcinoma (READ). **(C)** Kaplan-Meier curve of cumulative survival of COAD and READ cohorts in different categories of EGFR and c-MET associated with CAF infiltration. The ^high^CAF+^high^c-Met/EGFR cohort had a low overall survival rate.

We explored associations of C-met/EGFR expressions with infiltrating immune cells (CD4^+^ T cells, B cells, CD8^+^ T cells, neutrophils, dendritic cells, and macrophages) in CRC tissues (Figure 2A, Supplementary Table 1). Our results revealed positive correlations of EGFR expression with infiltration of macrophages, dendritic cells, CD4^+^ T cells, CD8^+^ T cells, neutrophils, and B cells. The correlation values were *r* = 0.173–0.537, *p* < 0.0001 in COAD and *r* = 0.191–0.3860, *p* < 0.01 in READ. c-MET was positively correlated with various immune cells; *r* = 0.185–0.259, *p* < 0.001 in COAD and *r* = 0.090–0.325 *p* < 0.05 in READ. Furthermore, EGFR was negatively correlated with tumor purity in COAD (*r* = -0.0716) and READ (*r* = -0.18717, *p* = 0.026802), while MET exhibited no significant correlations with tumor purity in COAD (*r* = 0.01029, *p* = 0.836045) and READ (*r* = 0.017459, *p* = 0.83777). Collectively, the above results indicate that c-MET/EGFR expressions were correlated with immune infiltration in COAD and READ (Figure 2A). Furthermore, we used the abundances of six immune cells and expression levels of c-MET/EGFR to construct univariate and multivariate Cox regression models. The univariate analysis indicated that higher infiltration levels of CD4^+^ T cell, macrophages, neutrophils, and dendritic cells were correlated with poor survival outcomes in COAD (Supplementary Figure 1), while low infiltration levels of CD8^+^ T cell, B cells, macrophages, neutrophils, and dendritic cells were correlated with poor survival outcomes in READ. A multivariate Cox regression model revealed that lower infiltration levels of CD4^+^ T cells (hazard ratio (HR) = 0.008, *p* = 0.015) were risk factors for COAD (Table 1).

**TABLE 1:** Multivariate Cox regression analysis of immune infiltration cells in COAD and READ.

	Colon adenocarcinoma				
Imm. Cell	Coef	HR	95% CI_l	95% CI_u	*p* value
B_cell	1.081	2.947	0.031	279.353	0.642
CD8_Tcell	−4.829	0.008	0.000	0.394	0.015
CD4_Tcell	−2.186	0.112	0.001	15.002	0.381
Macrophage	3.597	36.500	0.437	3,049.385	0.111
Neutrophil	−1.915	0.147	0.000	204.039	0.604
Dendritic	1.538	4.654	0.262	82.724	0.295
EGFR	0.269	1.309	0.955	1.793	0.094
MET	−0.136	0.873	0.644	1.183	0.380
	**Rectum Adenocarcinoma**				
		**Coef**	**HR**	**95% CI_l**	**95% CI_u**	***p* value**
B_cell	1.352	3.865	0.000	7,5468.261	0.789
CD8_Tcell	−8.946	0.000	0.000	7,934.891	0.328
CD4_Tcell	−4.442	0.012	0.000	3,042,847.483	0.653
Macrophage	4.165	64.385	0.000	38,262,895.774	0.539
Neutrophil	−5.099	0.006	0.000	99,534,652.430	0.671
Dendritic	5.968	390.575	0.042	3,600,096.976	0.200
EGFR	−0.488	0.614	0.289	1.305	0.205
MET	−0.065	0.938	0.489	1.796	0.846

We analyzed CAF correlations with expression profiles of c-MET/EGFR in CRC samples. We found that EGFR expressions were correlated with CAF infiltration in COAD (partial corr. = 0.281, *p* = 2.25e-06) and READ (partial corr. = 0.32, *p* = 2.11e-03). Similarly, c-Met expressions were positively correlated with CAF infiltration in COAD (partial corr. = 0.139, *p* = 4.91e-03) and READ (partial corr. = 0.214, *p* = 1.14e-02) (Figure 2B). To evaluate the prognostic relevance of these associations, we classified the cohorts into four groups; ^low^CAF+^low^c-Met/EGFR, ^low^CAF+^high^c-Met/EGFR, ^high^CAF+^low^c-Met/EGFR, and ^high^CAF+^high^c-Met/EGFR, and we found that the ^high^CAF+^high^c-Met/EGFR cohort had a low overall survival rate (Figure 2C).

### Genetic Alterations of Tyrosine-Protein Kinase/Epidermal Growth Factor Receptor are Associated With Poorer Prognoses of Colorectal Cancer Patients

**FIGURE 3:**
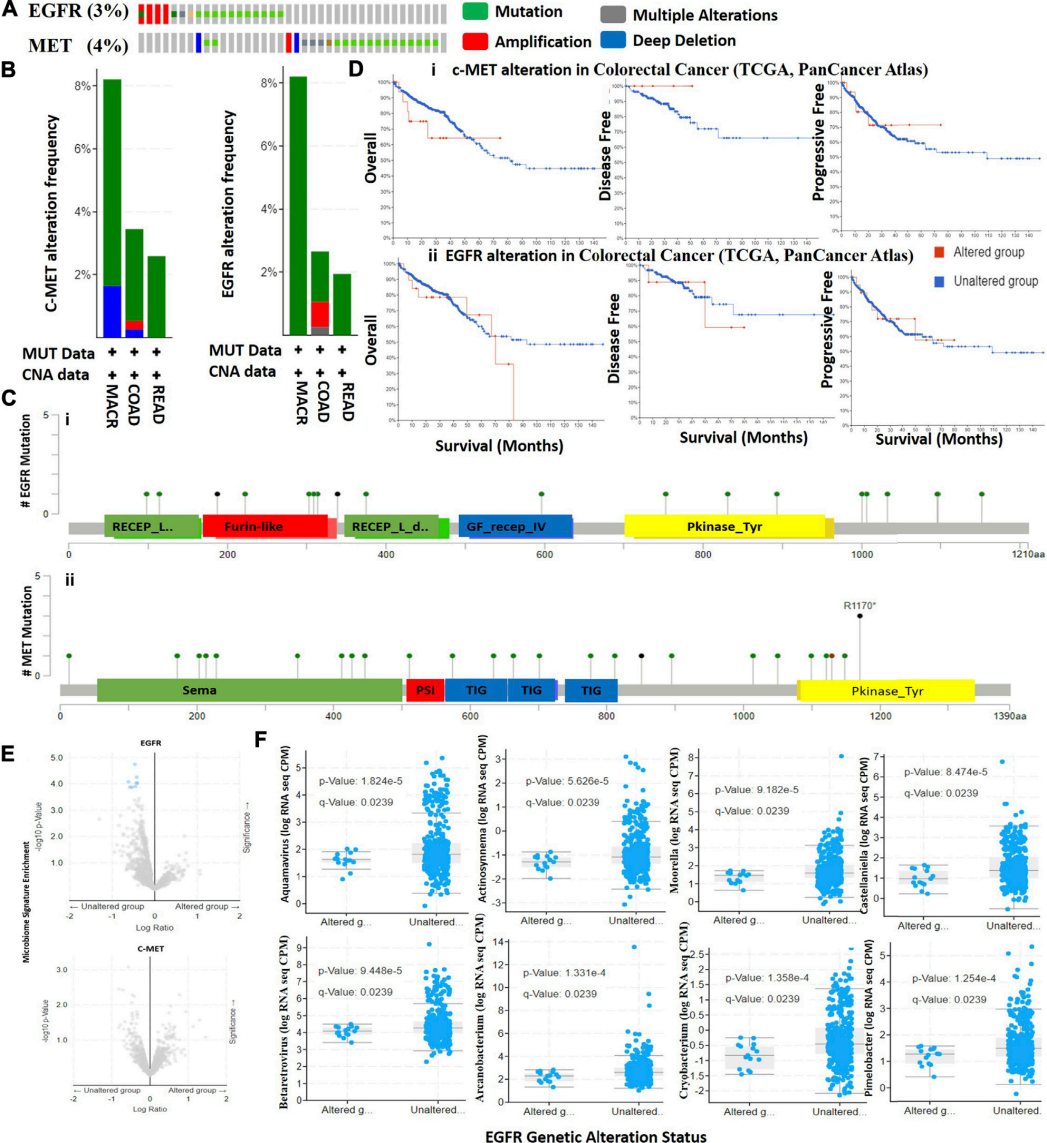
Genetic alterations in c-MET/epidermal growth factor receptor (EGFR) are associated with poor prognoses of colorectal cancer cohorts. **(A)** Total frequency of c-MET/EGFR genetic alterations in colorectal cancer patients. **(B)** Frequencies and types of c-MET/EGFR genetic alterations in colorectal cancer patients. COAD, colon adenocarcinoma; READ, rectum adenocarcinoma; MACR, mucinous adenocarcinoma of the colon and rectum. **(C)** Lollipop plot of c-MET/EGFR mutation types in colorectal cancer patients. Mutations are color-coded as missense, truncating, and in-frame mutations. **(D)** Kaplan-Meier plot of overall survival, disease-free survival, and disease progressive survival of colorectal cancer cohorts with c-MET **(i)** and EGFR **(ii)** genetic alterations. **(E)** Scatterplot of significance levels of enriched microbiome signatures in EGFR **(i)** and c-MET **(ii)** altered and non-altered colorectal cancer cohorts. **(F)** Scatterplot showing differential RNA expressions of microbiome signatures between EGFR altered and non-altered cohorts.

We mined TCGA PanCancer Atlas dataset for c-MET/EGFR genetic alteration data and evaluated the prognostic relevance of alterations in CRC patients. We found that 3.0 and 4.0% of the 594 CRC patients (63.6% of COAD, 26.1% of READ and 10.3% of mucinous adenocarcinoma (MUAD)) in the dataset respectively harbored genetically altered EGFR and c-MET (Figure 3A). Alterations in EGFR were mainly mutations (2.36%), amplifications (0.51%), and multiple alterations (0.17%), while alterations in c-MET were mutations (3.2%), amplifications (0.17%), and deep deletions (0.34%) (Figure 3B). By stratifying the mutations, we found 19 mutations consisting of 17 missense and two truncating mutations of EGFR, while c-MET mutations were stratified into 22 missense mutations, four truncations (all nonsense mutations; R1170* and S851*), and one in-frame deletion (I1130del) (Figure 3C). A comparative survival analysis indicated that CRC patients with genetic alterations of c-MET/EGFR exhibited shorter overall survival (OS), DFS, and progressive-free survival than cohorts without c-MET/EGFR alterations (Figure 3D).

### Distinct Microbiome Signatures are Biomarkers of Tyrosine-Protein Kinase/Epidermal Growth Factor Receptor Genetic Alterations in Colorectal Cancer

We analyzed RNA sequencing data of microbiome signatures between CRC cohorts with altered c-MET/EGFR and non-altered c-MET/EGFR. We found significantly higher levels of eight microbiome signatures including Aquamavirus (*p* = 1.82E-05), Actinosynnema (*p* = 5.63E-05), Castellaniella (*p* = 8.47E-05), Moorella (*p* = 9.18E-05), Betaretrovirus (*p* = 9.45E-05), Pimelobacter (*p* = 1.25E-04), Arcanobacterium (*p* = 1.33E-04), and Cryobacterium (*p* = 1.36E-04) in EGFR non-altered cohorts compared to altered cohorts (Figures 3E,F, Supplementary Table 2). However, no significant differences were detected in microbiome signatures between c-MET altered and non-altered cohorts (Figure 3F). Collectively, this study suggests that microbiome signatures were lost in CRC cohorts with genetically altered EGFR; thus, they could possibly serve as novel predictors of EGFR alterations in CRC patients.

### Genetic Alterations of Tyrosine-Protein Kinase/Epidermal Growth Factor Receptor in CRC Co-Occurred With Other Gene Alterations and are Associated With Overexpression of mRNAs of Some Cancer Hallmark Proteins

**FIGURE 4:**
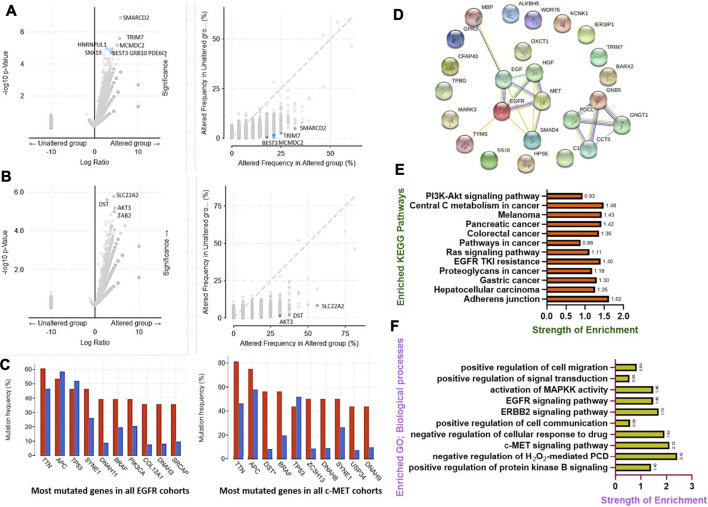
Genetic alterations of c-MET/epidermal growth factor receptor (EGFR) in colorectal cancer co-occurred with other gene alterations and were associated with overexpressions of mRNAs of some cancer hallmark proteins. Enrichment frequencies of gene alteration co-occurrences with c-MET/EGFR alterations in colorectal cancer cohorts. Scatterplot of the significance and frequencies of enriched co-occurring gene alterations in **(A)** EGFR and **(B)** c-MET altered and non-altered cancer cohorts. **(C)** Bar plot showing the top 10 most frequently mutated genes in c-MET/EGFR altered and non-altered cancer cohorts. **(D)** Protein-protein interaction (PPI) network, **(E)** enriched KEGG pathways, and **(F)** biological processes of overexpressed mRNAs in c-MET/EGFR altered colorectal cancer cohorts.

We analyzed the frequencies of the co-occurrence of gene alterations with c-MET/EGFR genetic alterations, and found the co-occurrence of genetic alterations in a total of 19,434 genes, enriched in both c-MET/EGFR altered and non-altered cohorts (Figures 4A, B). Genes with significantly (all *p* < 10^−4^) enriched genetic alteration co-occurrences with EGFR alterations included *SMARCD2*, *TRIM7*, *MCMDC2*, *HNRNPUL1*, *BEST3*, *GRB10*, *PDE6C*, and *SNX19*, while *SLC22A2*, *DST*, *AKT3*, and *TAB2* were the only significantly (all *p* < 10^−4^) enriched gene alterations that co-occurred with c-MET alterations (Supplementary Table 3). However, *TTN*, *APC*, *TP53*, *SYNE1*, *DNAH11*, *BRAF*, *PIK3CA*, *COL12A1*, *DNAH3*, and *SRCAP* had the highest mutation frequencies in both EGFR altered and non-altered cohorts, while *TTN*, *APC*, *DST*, *BRAF*, *TP53*, *ZC3H13*, *DNAAH8*, *SYNE1*, *USP34*, and *DNAH9* were the most frequently mutated genes associated with c-MET altered and non-altered cohorts (Figure 4C).

Furthermore, we found that genetic alterations of EGFR were associated with overexpressions of mRNAs of a number of proteins in CRC patients. The top ten overexpressed (*p* = 7.47E-05–2.97E-06) mRNAs in EGFR altered cohorts compared to non-altered cohorts were *TRIM7*, *BARX2*, *CFAP43*, *TPBG*, *HPSE*, *GNB5*, *MARK3*, *FUT8-AS1*, *SS18*, and *KCNK1*, while *SMAD4*, *MBP*, *IER3IP1*, *OXCT1*, *ALKBH5*, *C18ORF25*, *WDR76*, *TYMS*, *LINC00909*, and *GPR3* were overexpressed in c-MET altered CRC cohorts (Supplementary Table 4).

We further constructed PPI networks of overexpressed mRNAs in c-MET/EGFR altered cohorts (Figure 4D) and performed enrichment analyses. We found that overexpressed mRNAs were enriched in several KEGG pathways associated with CRC development, progression, and drug resistance. The top enriched pathways (with a strength of enrichment (SOE) of >1 and *p* < 0.05) included cell adherent junctions, EGFR tyrosine kinase inhibitor resistance, central carbon metabolism, CRC, pancreatic cancer, hepatocellular carcinoma, gastric cancer, and melanomas (Figure 4E), while enriched GOs (with a SOE of >1 and *p* < 0.05) included the c-MET, ERBB2, and EGFR signaling pathways, MAPKK activation, and negative regulation of cellular responses to drugs (Figure 4F).

### DNA-Methylation and Somatic Copy Number Alterations of Tyrosine-Protein Kinase/Epidermal Growth Factor Receptor are Associated With Immune Infiltration, Dysfunctional T Cell Phenotypes, and Poor Prognoses of Colorectal Cancer Patients

**FIGURE 5:**
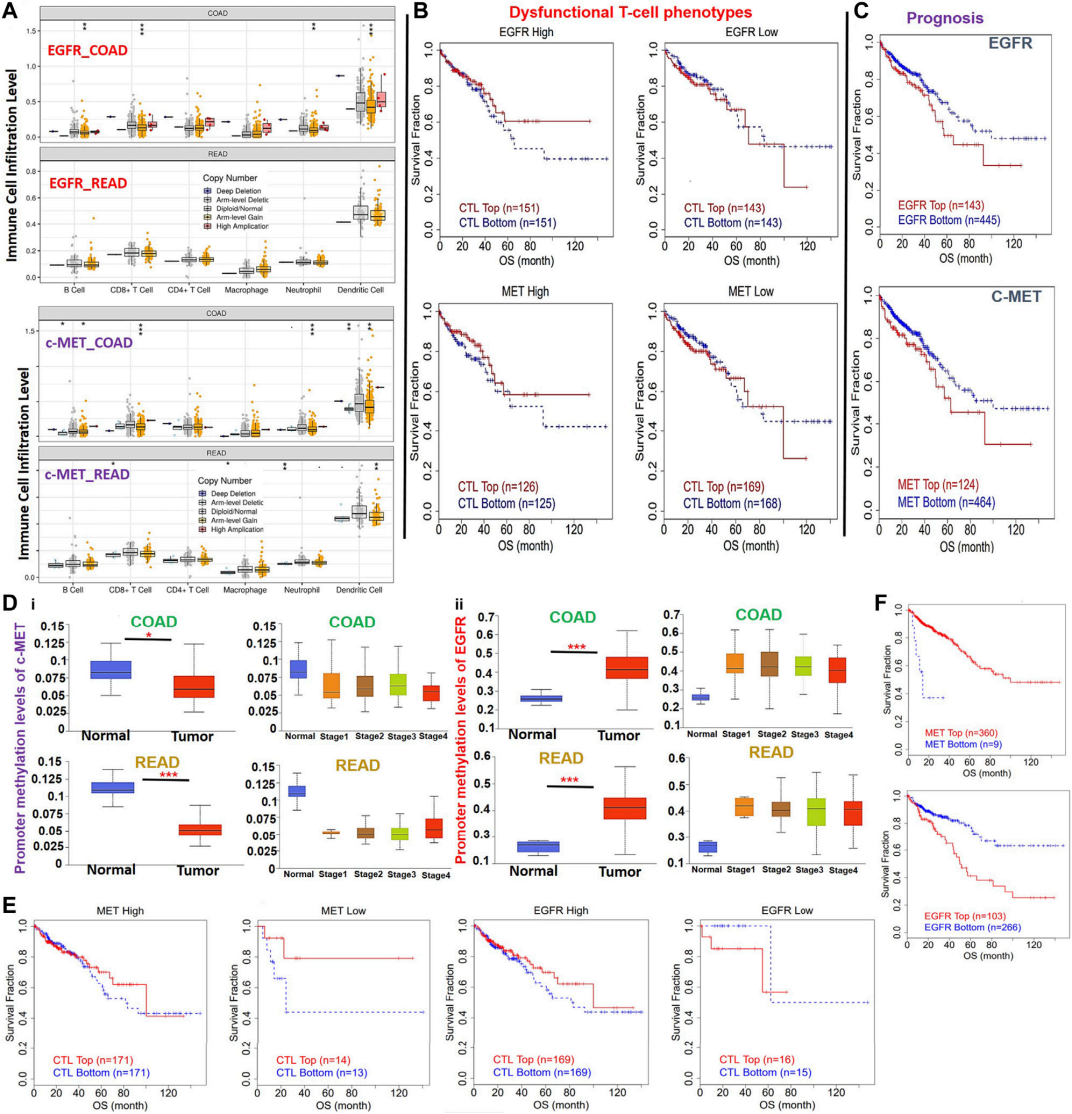
DNA-methylation and copy number alterations (CNAs) of c-MET/epidermal growth factor receptor (EGFR) are associated with dysfunctional T cell phenotypes and poor prognoses of colorectal cancer patients. **(A)** Box plots showing tumor immune infiltration levels in colorectal cancer with different somatic CNAs of c-Met/EGFR. The infiltration abundance in every somatic CNA category was compared to the diploid/normal ratio. *p < 0.05; **p < 0.01; ***p < 0.001. **(B)** Kaplan-Meier plot of the independent effects of c-MET/EGFR CNAs on dysfunctional T cell phenotypes. **(C)** Kaplan-Meier curve of the overall survival of patient with high or low somatic CNAs of c-MET/EGFR. **(D)** Bar plot showing differential methylation levels of c-MET/EGFR between normal tissues and colorectal tumors **(i)** and different tumor stages **(ii)**. **(E)** Kaplan-Meier plot of the independent effects of c-MET/EGFR methylation on dysfunctional T cell phenotypes. **(F)** Kaplan-Meier curve of the overall survival of patients with a hypo- or hypermethylation status of c-MET/EGFR.

We also analyzed associations of different somatic CNAs, such as arm-level gains, high amplifications, deep deletions, arm-level deletions of c-MET/EGFR and immune cell infiltration in CRC. We found that arm-level gains of EGFR were associated with B cell (*p* < 0.005), CD8^+^ T cell (*p* < 0.001), neutrophil (*p* < 0.005), and dendritic cell infiltration in COAD, and macrophage (*p* < 0.005) infiltration in READ. Arm-level gains of c-MET were positively correlated with CD8^+^ T cell, B cell, neutrophil, and dendritic cell infiltration in colon cancer and dendritic cells infiltration in READ, while deep deletions of c-MET were associated with CD8^+^ T cell and neutrophil infiltration of tumors in READ (Figure 5A). Furthermore, CNAs of both c-MET and EGFR were associated with dysfunctional T-cell phenotypes (Figure 5B) and poor prognoses (Figure 5C) of CRC patients. c-MET was found to be hypomethylated, while EGFR was hypermethylated in CRC (Figure 5D). These methylation statuses of c-MET and EGFR in CRC were also consistently associated with the tumor stage (Figure 5D), dysfunctional T cell phenotypes (Figure 5E), and shorter OS of CRC patients (Figure 5F). Collectively, these findings indicated that the DNA-methylation and somatic CNAs particularly the arm-level gains of c-MET/EGFR were associated with immune infiltration, dysfunctional T cell phenotypes, and poor prognoses of CRC patients.

### NSC777205 and NSC777207 Displayed Desirable Characteristic of a Good Drug Candidate and Exhibited *In Silico* Cytotoxic Activities Against Colon, Brain, Lung, and Bone Cancer Cell Lines

**FIGURE 6:**
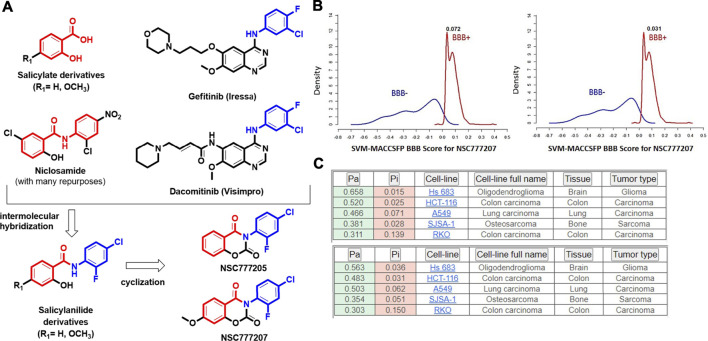
**(A)** Synthesis protocols for NSC777205 (3-(4-chloro-2-fluorophenyl)-2H-benzo[e [1,3]-oxazine-2,4(3H)-dione) and NSC777207 (3-(4-chloro-2-fluorophenyl)-7-methoxy-2H-benzo[e][1,3]-oxazine-2,4(3H)-dione), which were obtained via intermolecular hybridization and condensation reactions between salicylic acid (R_1_ = H) or 2-hydroxy-4-methoxybenzoic acid (R_1_ = OCH_3_) and 2,4-disubstituted aniline moieties of gefitinib to produce salicylanilides which were further respectively cyclized to produce NSC777205 (when R_1_ = H) and NSC777207 (when R_1_ = OCH_3_). Cyclization occurred in the presence of methyl chloroformate and pyridine, while hybridization occurred in the presence of tetrahydrofuran (THF). **(B)** Blood-brain barrier (BBB) permeation curves of NSC777205 and NSC777207 as measured by the support vector machine (SVM) and LiCABEDS algorithms of the BBB prediction server. **(C)** PASS predicted cytotoxic activities of NSC777205 and NSC777207 against various cancer cell lines.

NSC777205 (3-(4-chloro-2-fluorophenyl)-2H-benzo[e][1,3]oxazine-2,4(3H)-dione) and NSC777207 (3-(4-chloro-2-fluorophenyl)-7-methoxy-2H-benzo[e][1,3]-oxazine-2,4(3H)-dione) were obtained at high purity (>95%) via condensation reactions between salicylic acid and the 2,4-disubstituted aniline moieties of gefitinib to produce a salicylanilide which was further cyclized to the 3-phenyl-2H-benzo[e][1,3]-oxazine-2,4(3H)-dione derivatives, NSC777205 and NSC777207, as summarized in Figure 6A. Our general *in silico* analysis of drug-like and toxicity characteristics of NSC777205 and NSC777207 suggested that both compounds exhibited suitable physicochemical properties (lipophilicity, polarity, flexibility, solubility, saturation, molecular weight, and other properties) of a good drug candidate. The compounds were both lead-like and satisfied Lipinski's rules for drug likeness candidates. Furthermore, our *in silico* analysis suggested that both compounds can be easily absorbed in the gastrointestinal tract and are well permeant to the blood-brain barrier (BBB) especially NSC777205 which demonstrated 2-fold higher permeation of the BBB than NSC777207 based on *in silico* estimation (Figure 6B; Table 2). Estimates of the acute toxicity in rats revealed high LD_50_ values for NSC777205 and NSC777207 from oral, intravenous, intraperitoneal, and subcutaneous administration, while bioaccumulation factor (log10) and other markers of eco-toxicity also identified both compounds as having low toxicities (Table 2). Collectively, these findings suggested that NSC777205 and NSC777207 exhibited desirable characteristic of a drug candidates and can safely be employed for acute administration via oral, intravenous, intraperitoneal, and subcutaneous routes. Our *in silico* cytotoxic analysis suggested that both NSC777205 and NSC777207 exhibited cytotoxic activities against colon (HCT-116 and RKO), brain (Hs683), lung (A549), and bone (SJSA-1) cancer cell lines, with NSC777205 demonstrating higher activities (Pa = 0.31–0.658) than NSC777207 (Pa = 0.303–0.565) against all cell lines (Figure 6C).

**TABLE 2:** Drug-like and toxicity characteristic of NSC777205 and NSC777207.

Properties	NSC777205	NSC777207	Reference value
Formula	C_14_H_7_ClFNO_3_	C_15_H_9_ClFNO_4_	—
M.W(g/mol)	291.66	321.69	150–500
R-bonds	1	2	0–9
H-bond ACC.	4	5	0–10
H-bond DON.	0	2	0–5
Molar Refractivity	73.05	79.54	40–130
TPSA (Å^2^)	52.21	61.44	20–130
Fraction Csp3	0.00	0.07	0.25 ∼ <1
Log Po/w (XLOGP3)		3.46	−0.7 to 5
Consensus Log Po/w	3.15	3.29	≤3.5
Log S (ESOL)	−4.37	−4.42	0–6
Drug-likeness (Lipinski rule)	YES 0 violation	YES 0 violation	MLOGP ≤ 4.15, M.W ≤ 500, H-bond ACC ≤ 10, H-bond DON ≤ 5
Lead-likeness	YES	YES	XLOGP3 ≤ 3.5, M.W ≤ 350, R-bonds ≤ 7
Bioavailability Score	0.55	0.55	>0.1 (10%)
BBB-permeation (LogBB)	High (0.631)	(-0.400)	BBB+ ≥ 0.3, BBB− < −1,
BBB-permeation (SVM_MACCSFP)	0.072	0.031	≥0.02
Synthetic accessibility	3.06	3.08	1 (very easy) to 10 (very difficult).
Route of adm.			
Intraperitoneal	357.300 (OECD:4)	224.500 (OECD:4)	
Intravenous	116.800 (OECD:4)	201.500 (OECD:4)	
Oral	943.300 (OECD:4)	591.800 (OECD:4)	
Subcutaneous	2161.000 (OECD:5)	740.800 (OECD:4)	
Bioaccumulation factor Log10(BCF)	1.462	1.125	<2: low, 2 ≤ 3: Moderate, 3–3.7: High, >3.7: Very high
Daphnia magna LC50 −Log_10_ (mol/L)	6.421	6.599	
Fathead Minnow LC_50_Log_10_ (mmol/L)	−1.931	−2.108	
Tetrahymena pyriformis IGC50 -Log_10_ (mol/L)	1.401	1.453	

R-bond, Num. rotatable bonds; H-bond ACC, Num. H-bond acceptors; H-bond DON, H-bond donors; TPSA, topological polar surface area; BBB, Blood brain barrier; IP, Intraperitoneal; IV, Intravenous; SC, Subcutaneous.

### NSC777205 and NSC777207 Exhibited Wide Spectra of Antiproliferative Activities and Selective Cytotoxic Preferences for Melanoma, Renal, Central Nervous System, Colon, and NSCLC Cell Lines *In Vitro*

We initially screened NSC777205 and NSC777207 for anticancer activities against the full NCI-60 panel of human tumor cell lines. Interestingly, we found that with single-dose (10 μM) treatment, both NSC777205 and NSC777207 demonstrated antiproliferative activities against all of the NCI-60 cell line panels of breast, prostate, renal, ovarian, colon, melanoma, CNS, leukemia, and non-small cell lung cancers, with NSC777205 demonstrating higher activities than NSC777207. In addition, most of the leukemia (cell death (CD) = 0.77–10.28%), NSCLC (CD = 4.2–50.81%), CNS (CD = 013.71%), melanoma (CD = 0.57–46.24%) renal (CD = 7.01–30.91%), breast (CD = 10.48%), prostate, and ovarian cancer cell lines demonstrated some cytotoxic response to NSC777205 and NSC777207 single-dose treatment, while panels of colon cancer cell lines were completely insensitive to the cytotoxic activity of the compounds at 10 μM. Collectively, these studies demonstrated that both NSC777205 and NSC777207 exhibited a wide spectrum of anticancer activities but demonstrated selective and exclusive antiproliferative activities against all panels of colon cancer cell lines (Supplementary Figure 2).

**FIGURE 7:**
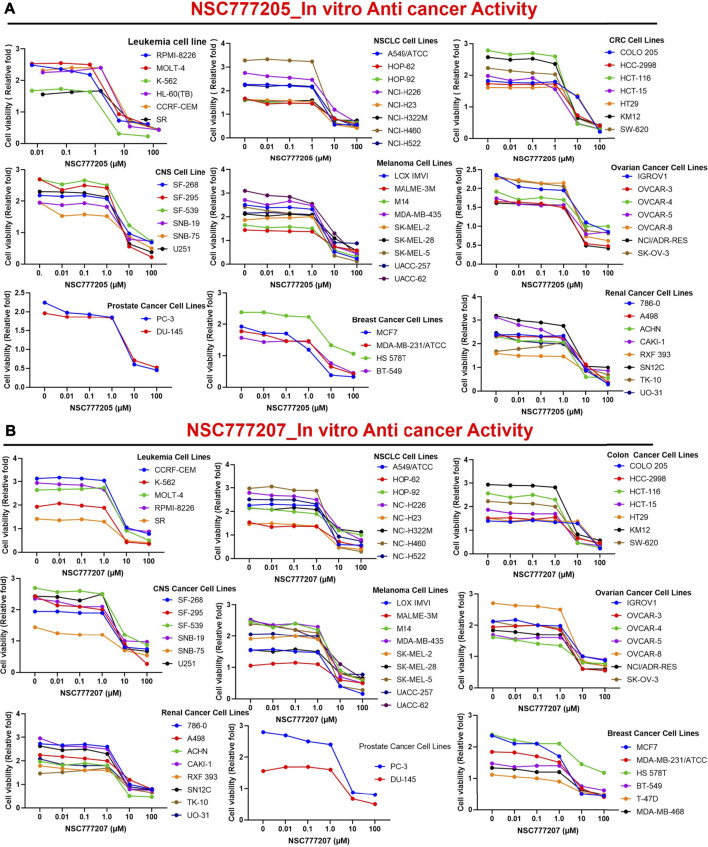
*In vitro* anticancer activities of NSC777205 and NSC777207 against the NCI-60 human cancer cell lines. Dose-response curves of NCI-60 human cancer cell lines to **(A)** NSC777205 and **(B)** NSC777207 treatment.

**FIGURE 8:**
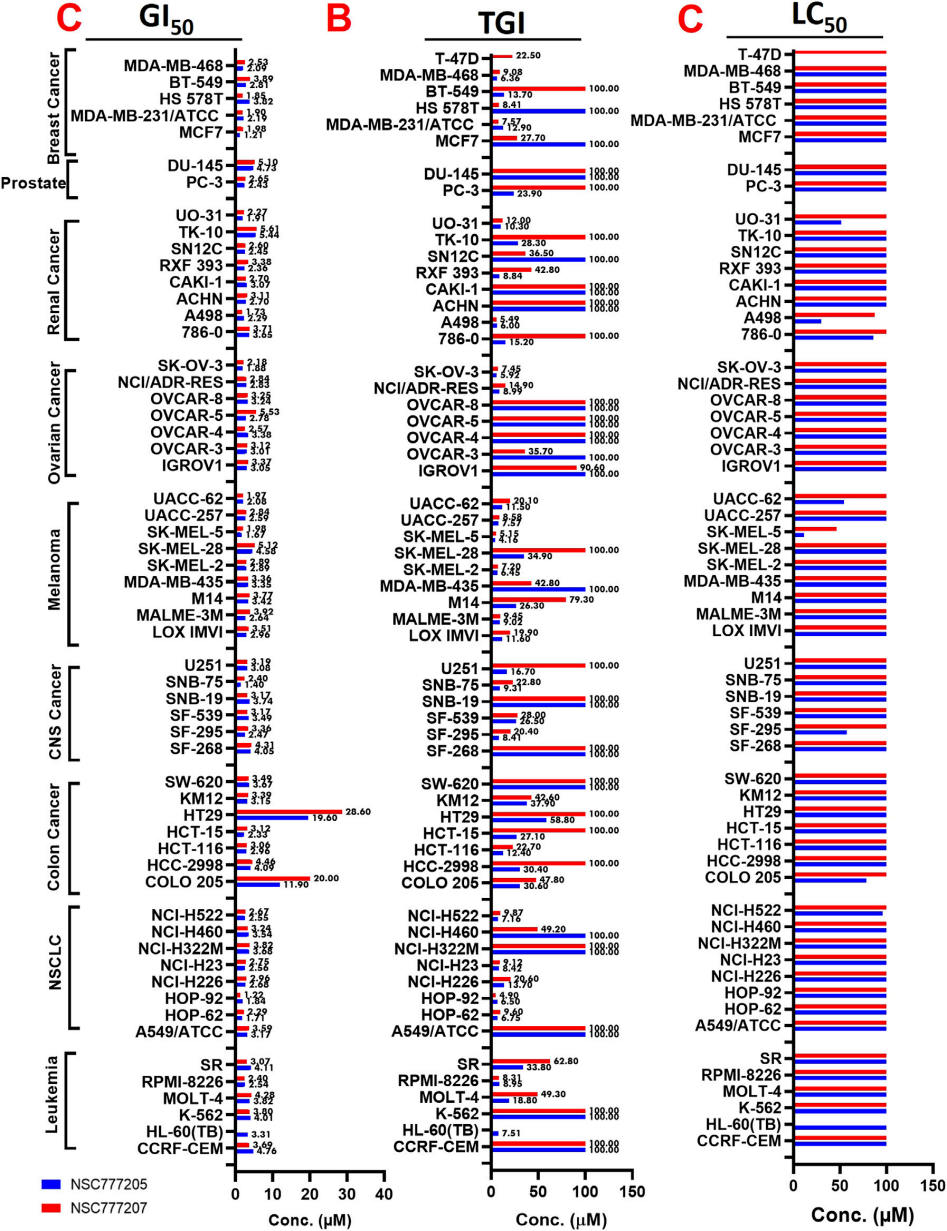
Heat map showing the (C) GI_50_ (D) TGI and (E) LC_50_ value of NSC777205 and NSC777207 against the NCI-60 human cancer cell lines.

With five-dose screening, NSC777205 and NSC777207 demonstrated dose-dependent cytotoxic effects against the panel of NCI-60 human tumor cell lines (Figures 7A, B). With the exception of HT29 and COLO 205 (GI_50_ = 11.90–20 µM), both NSC777205 and NSC777207 exhibited high anticancer activities with GI_50_ concentrations of <6 µM against all cell line panels (Figure 8A). Total cell growth inhibitory (TGI) concentrations of the compounds revealed that NSCLC (1.71–3.68 µM), leukemia (7.51–33.80 µM), CNS (7.51–33.80 µM), melanoma (4.196–34.90 µM), and colon (12.40–58.80 µM) cancer cell lines were the most sensitive to NSC777205 treatment, while a lower but similar trend of anticancer activity against the panel of cell lines was also observed for NSC777207 (Figure 8B). Furthermore, as revealed by LC_50_ values, cytotoxic activities of NSC777205 were more pronounced against the 786–0 (LC_50_ = 85.70 µM), A498 (LC_50_ = 29.60 µM), and UO-31 (LC_50_ = 51.10 µM) renal cancer cell lines; the SK-MEL-5 (LC_50_ = 11.30 µM) and UACC-62 (LC_50_ = 54.10 µM) melanoma cell lines; the SF-295 (LC_50_ = 57.40 µM) CNS cell line; the COLO 205 (LC_50_ = 78.40 µM) colon cancer cell line; and the NCI-H522 (LC_50_ = 95.70 µM) NSCLC cell line (Figure 8C). Collectively, the anticancer study revealed that NSC777205 and NSC777207 exhibited a wide spectrum of antiproliferative activities and selective cytotoxic preferences for renal cancer, melanoma, CNS cancer, colon cancer, and NSCLC cell lines with NSC777205 demonstrating higher potencies than NSC777207.

### Tyrosine-Protein Kinase/Epidermal Growth Factor Receptor/Phosphatidylinositol 3-Kinase/Mammalian Target of Rapamycin Signaling Pathways are Implicated in the Anticancer Activities of NSC777205 and NSC777207

In order to adequately identify potential drug targets of our compounds, we used three different *in silico* drug target identification algorithms and also the DTP-COMPARE algorithm. Using PharmMapper, c-MET and EGFR were both identified as potential targets for NSC777205 and NSC777207. The pharmMapper algorithm identified c-MET as potential targets for NSC777205 and NSC777207 by pharmacophore mapping of one hydrogen bond and four hydrophobic interactions, with normalized fit scores of 0.5876 and 0.5884, while EGFR was mapped with NSC777205 and NSC777207 by two hydrophobic reactions and two hydrogen interactions with normalized fit scores of 0.6981 and 0.6986 (Supplementary Table 5). In addition, the PI3K regulatory subunit alpha, several MAPKs, and several serine/threonine-protein kinases were identified as potential targets for NSC777205 and NSC777207 (Supplementary Table 5). The PASS prediction server also identified c-MET and EGFR as potential targets for NSC777205 and NSC777207 with pa>0.3 and pi<0.06. In addition, inhibition of MAPK, inositol 1,4,5-trisphosphate 3-kinase, and growth factors among other activities were predicted for NSC777205 and NSC777207 (Supplementary Table 6). However, SwissTargetPredition for NSC777207 returned serine/threonine-protein kinases, mTOR, several subunits of PI3K, MAPK, and ErbB-2 among others, while AKT, EGFR, mTOR, several subunits of PI3K, and growth factors were predicted for NSC777205 (Supplementary Table 7). Altogether, c-MET, EGFR, PI3K, and mTOR were commonly identified targets of NSC777205 and NSC777207 by the three drug target identification algorithms we employed. Furthermore, results of the DTP’s COMPARE algorithm indicated that the anticancer fingerprints of NSC777205 and NSC777207 showed strong correlations (*p* = 0.58–0.86, common cell line count (CCLC) = 47–58) with fingerprints of some NCI synthetic compounds (Table 3). These synthetic compounds are small molecules with molecular weights ranging 277.8–559.5 g/mol. The COMPARE analysis of NSC777205 and NSC777207 anticancer fingerprints with NCI investigational drugs revealed strong associations (CCLC = 55–58, *p* = 0.3–0.61) with a number of c-MET/EGFR/PI3K/AKT/mTOR inhibitors, including JNJ38877618, MTX211, SGX-523, altiratinib, TAS-6417, voxtalisib, PI-103 HCL, AMG-458 (c-MET), CC115, and others (Table 4). In addition, the COMPARE analysis identified MET, EGFR, PIK3R2, RPS6KB1, CDK6, IGHMBP2, and ErbB2 as some of the major targets that were correlated with the anticancer fingerprints of NSC777205 and NSC777207 (Table 4). Collectively this study provides a hypothesis implicating C-MET/EGFR/PI3K/AKT/mTOR signaling pathways in the anticancer activities of NSC777205 and NSC777207.

**TABLE 3:** NCI synthetic compound correlation with anticancer fingerprint of NSC777205 and NSC777207.

Rank	*P*	CCLC	M.W (g/mol)	NCI-ID	Target descriptor
	NSC777205				
1	0.78	55	413.14	777213	3-(2-fluoro-4-iodophenyl)-7-methoxy-2H-benzo[e] [1,3]oxazine-2,4(3H)-dione
2	0.72	56	307.3	50681	3-Hydroxy-2-naphtho-o-phenetidide
3	0.72	56	277.8	37188	2-Naphthalenecarboxamide, 3-hydroxy-N-(2-methylphenyl)-
4	0.72	58	327.12	178296	NICLOSAMIDE(USAN)
5	0.7	58	451.6	766722	(2Z)-3-(1H-Benzimidazol-2-yl)-6-hexyl-2-[(2-methylphenyl)imino]-2H-chromen-7-ol
6	0.68	56	361.29	765598	N-(2,4-difluorophenyl)-2',4'-difluoro-4-hydroxybiphenyl-3-carboxamide
7	0.63	58	317.4	775727	3-Isobutyl-9,10-dimethoxy-1,3,4,6,7,11b-hexahydro-2H-benzo[a]quinolizin-2-one(T2839)
8	0.61	44	310.4	653264	2-[1-(Benzenesulfonyl)indol-3-yl]propanenitrile
9	0.6	58	297.7	12969	2-Naphthalenecarboxamide, N-(3-chlorophenyl)-3-hydroxy-
10	0.59	47	416.4	640519	3'-Carboxypropionyl-(Z)-combretastatin A-4
11	0.47	58	376.31	765690	(4-(6-(2,4-Difluorophenyl)-2,4-dioxo-2H-benzo[e][1,3]-oxazin-3(4H)-yl) benzonitrile
12	0.58	58	372.2	367089	NAPHTHOL AS-BI
13	0.58	56	357.8	50687	5'-Chloro-3-hydroxy-2',4'-dimethoxy-2-naphthanilide
**Rank**	**NSC777207**				
1	0.86	58	327.12	178296	NICLOSAMIDE(USAN)
2	0.84	56	307.3	50681	3-Hydroxy-2-naphtho-o-phenetidide
3	0.81	55	413.14	777213	3-(2-fluoro-4-iodophenyl)-7-methoxy-2H-benzo[e][1,3]oxazine-2,4(3H)-dione
4	0.77	58	451.6	766722	(2Z)-3-(1H-Benzimidazol-2-yl)-6-hexyl-2-[(2-methylphenyl)imino-2H-chromen-7-ol
5	0.75	56	277.8	37188	2-Naphthalenecarboxamide, 3-hydroxy-N-(2-methylphenyl)-
6	0.73	58	317.4	775727	3-Isobutyl-9,10-dimethoxy-1,3,4,6,7,11b-hexahydro-2H-benzo[a]quinolizin-2-one(T2839)
7	0.68	58	290.4	240579	4-(2-(4-(Dimethylamino)phenyl)vinyl)-8-quinolinol
8	0.68	50	382.4	709579	N-(3-Fluorophenyl)-6-hexyl-7-hydroxy-2-iminochromene-3-carboxamide
9	0.68	58	269.12	757391	DICHLOROPHENE
10	0.65	54	559.5	642198	Antineoplastic-642198

P, Pearson’s correlation coefficient; CCLC, Common cell lines count; MW, molecular weight.

**TABLE 4:** NCI investigational drugs and molecular targets correlation with anticancer fingerprint of NSC777205 and NSC777207.

	Molecular targets			Investigational drug			
Rank	*P*	CCLC	Target	*P*	CCLC	Targets	Mechanism of action
	NSC777205								
1	0.35	51	IGHMBP2	0.55	58	PI-103 HCL	PIK3CB inhibitor
2	0.34	55	CG2572	0.50	57	S1038	PI3K inhibitor
3	0.33	56	GRM3	0.48	58	FH535	Wnt/β-catenin inhibitor
4	0.31	57	CDK6	0.48	57	PALOMID-529	TORC1/TORC2 inhibitor
5	0.3	57	MET	0.44	57	BMS986195	inhibitor of BTK,
6	0.31	57	RPS6KB1	0.43	58	CC115	mTOR/PI3K inhibitor
7	0.28	56	CTTN	0.43	55	PNU-74654	CTNNB1-catenin beta 1 inhibitor
8	0.27	54	MOS	0.42	58	CS-1202	PIK3CA inhibitor
9	0.24	57	EGFR	0.41	58	PP242	mTOR inhibitor
10	0.24	54	SLC25A13	0.41	57	TENALISIB	PI3K inhibitor
11	0.24	45	CG2460	0.32	56	TELATINIB	VEGFR/PDGFα/c-Kit Inhibitor
12	0.22	52	PIK3R2	0.32	58	AMG-458 (C-MET)	Met (c-Met) kinase inhibitor
13	0.21	56	ERBB2	0.31	57	JNJ38877618	Met (c-Met) kinase inhibitor
14	0.20	56	TGFB2	0.31	57	TAS120	FGFR Inhibitor
15	0.19	57	CCND1	0.3	58	MTX211	EGFR Inhibitor
	**NSC777207**							
1	0.43	52	IGHMBP2	0.61	56	ROGARATINIB	FGFRs
2	0.36	56	CTTN	0.53	58	CC115	mTOR inhibitor
3	0.31	57	FGF3	0.52	58	CEP-32496	MEK
4	0.3	57	CCND1	0.49	58	GDC-0349	mTOR/MEK
5	0.24	55	CG2572	0.49	55	ASP-5878	FGFR
6	0.23	57	CDK6	0.49	56	NVP-BEZ235	PI3K/mTOR
7	0.21	57	MET	0.48	58	ALTIRATINIB	c-Met/HGFR inhibitor
8	0.18	57	RPS6KB1	0.47	58	10058-F4	c-Myc inhibitor
9	0.18	55	HBE1	0.46	58	PP242	mTOR/RET
10	0.14	57	BRCA2	0.46	57	VOXTALISIB	PI3K/mTOR Inhibitor
11	0.13	56	TGFB2	0.42	58	NVP-BGT226	PI3K/mTOR
12	0.13	57	EGFR	0.41	58	TAS-6417	EGFR
13	0.13	57	MYCN	0.4	57	PWT33597	PI3K/mTOR
14	0.12	51	RARB	0.38	58	SGX-523	c-Met
15	0.12	52	PIK3R2	0.38	58	MTX211	EGFR

P, Pearson’s correlation coefficient; CCLC, Common cell lines count; MW, molecular weight.

### Comparative Docking Profile of Tyrosine-Protein Kinase/Epidermal Growth Factor Receptor/Phosphatidylinositol 3-Kinase-Mammalian Target of Rapamycin Between NSC777205, NSC777207 and Clinical Inhibitors

**FIGURE 9:**
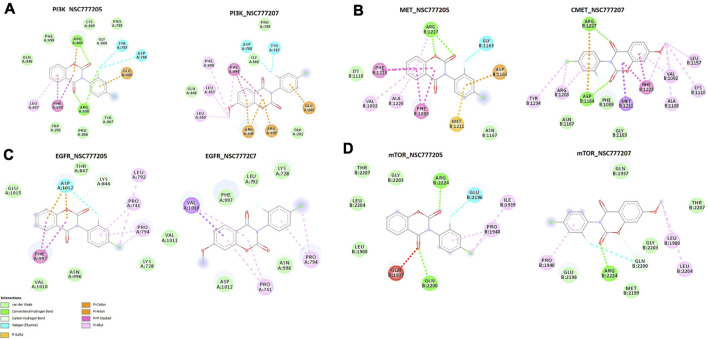
The two dimensional (2D) representations of the docking profiles of NSC777205 and NSC777207 with **(A)** PI3K (PDB:3APC), **(B)** c-MET (PDB: 3RHK), **(C)** EGFR (PDB: 5EDP), and **(D)** mTOR (PDB: 5FLC).

We used molecular docking to further understand the possible interactions of NSC777205 and NSC777207 with c-MET/EGFR/PI3K-mTOR. We found that NSC777205, NSC777207 and clinical inhibitors (used for comparison) docked well into the binding cavities of the receptors with respective binding affinities (ΔG) of −6.90, −6.80 and −6.80 Kcal/mol for EGFR; −8.95, 8.96 and −9.56 Kcal/mol for c-MET; −8.60, −7.90, and −9.0 Kcal/mol for PI3K; and −7.1, −7.6 and −9.2 Kcal/mol for mTOR (Table 5). The interactions of the ligands with the receptors (c-MET/EGFR/PI3K-mTOR) predominantly involved hydrogen bonds, hydrophobic interactions, various π-interactions, and Van der Waals forces (Figure 9; Supplementary Figure 3; Tables 5),

**TABLE 5:** Comparative docking profile of NSC777205, NSC777207 and clinical inhibitors of C-MET/EGFR/PI3K-mTOR.

	EGFR						c-MET					
	NSC777205	DIS (Ӑ)	NSC777207	DIS (Ӑ)	Gefitinib	DIS (Ӑ)	NSC777205	DIS (Ӑ)	NSC777207	DIS (Ӑ)	Crizotinib	DIS (Ӑ)
ΔG = (Kcal/mol)	−6.90		−6.80		−6.80		−8.9		−8.9		−9.5	
No. hydrophobic contact	6		4		5		5		6		6	
ConventionalH-bond					MET793	2.32	ARG1227	2.62	ARG1227	2.60		
							ARG1227	2.57	ASP1164	2.75		
C-H bond	LYS846	3.47			ASP800	3.71					ARG1208	3.69
					GLN791	3.50						
Halogen bond	ASP1012	3.56			ASP855	3.09	GLY1163	3.65				
π -anion	ASP1012	3.30					ASP1164	3.58	ASP1164	3.18	ASP1164	3.26
π -cation									ARG1227		ARG1227	
π –sulfur							MET1211					
π -alkyl	LEU792		PRO741		ALA743		VAL1092		LEU1157		ALA1108	
	PRO741		VAL1010		VAL726		ALA1226		VAL1092			
									LYS1110			
									ALA1108		LEU1157	
	PRO794		PRO794						ARG1208		LYS1110	
									TYR1234			
π -π T-shaped	PHE997											
π -π stacked							PHE1089		PHE1223		PHE1089	
							PHE1223					
π-sigma			VAL101		LEU844				MEY1211		PHE1223	
			0		LEU718						VAL1092	
Van der waal forces	VAL1010		VAL101		GLY719		LS1110		ASN1167		ARG1166	
			1ASP10		LYS745				PHE1089		MET1211	

			12		PHE723				GLY1163		ALA1226	
			PHE997,		MET790							
			LEU792		ARG841							
			LYS728		ASN842							
	ASN996		ASN996		THR854							
					LEU792							
					GLY796							

					PRO794							

					CYS797							

	LYS728										GLY1085	
	GLU1015										ILE1084	
	THR847										TYR1234	
Hydrophobic Interactions	PRO741	3.64	PRO794	3.76	LEU718	3.77	PHE1089,	3.75	PHE1089,	3.92	PHE1089	3.99
	, LEU792	3.72	PRO794	3.68	, LEU718	3.70	PHE1089,	3.49	VAL1092	3.64	VAL1092	3.93
	PRO794,	3.45	VAL101	3.60	, LEU718	3.90	VAL1092	3.12	VAL1092	3.92	ALA1108	3.83
	PHE997	3.89	0	3.48	VAL726	3.59	PHE1223	3.70	LYS1110	3.86	LYS1110	3.58
	PHE997,	3.68	ASP101		, VAL726	3.59	ALA1226	3.90	LEU1157	3.56	LEU1157	3.75
	ASP10121	3.48	2						PHE1223	3.69	PHE1223	3.96
	PI3K						mTOR					
	NSC777205_c-pi3k	DIS (Ӑ)	NSC777207_c-pi3k	DIS (Ӑ)	COPANLISIB	DIS (Ӑ)	NSC777205_mTOR	DIS (Ӑ)	NSC777207_mTOR	DIS (Ӑ)	DACTOLISIB	
ΔG=(Kcal/mol)	−8.6		−7.9		−9.0		−7.1		−7.6		−9.2	
No. hydrophobic contact	2		5		2		4		3		4	
ConventionalH-bond	ARG690	2.40			ARG690	2.61	ARG2224	2.12	ARG2224	2.22	GLN1937	2.19
	ARG690	2.52										
	ARG849	3.36			CYS869	2.28	GLN2200	2.86			ARG2224	2.35
	ARG849	2.94										
C-H bond	GLY868	3.23			TYR787	3.50			GLN2200	3.21		
					GLU852	3.63						
Halogen bond	TYR787	3.01	ASP788	3.45			GLU2196	3.54	GLN2200	3.45		
	ASP788	3.58	TYR787	3.04								
π -anion	GLU880		GLU880								ASP2145	4.82
π -cation	ARG849	3.77	ARG849	3.70	ARG849	3.20						
			ARG690									
π -alkyl	LEU657		PHE698		PRO866		ILE1939		LEU1900		PRO2146	
			LEU657		LYS298		PRO1940		LEU2204		LEU1900	
			LEU660						PRO1940		LEU2204	
π -π T-shaped					TRP201							
π -π stacked	PHE694	3.92	PHE694									
Amide-π stacked											LEU1936	
Van der waal forces	GLN846		GLN846		ASP861		THR2207		GLU2196		ALA1971	
	PHE698		TRP201		HIS295		LEU2204		MET2199		ILE1939	
	CYS869		GLY868		GLY868		LEU1900		GLY2203		GLU2196	
	PRO789		PRO789		MET842		GLY2203		THR2207		MET2199	
	TYR867				ILE870				GLN1937		GLN2200	
	PRO866				PHE898						GLY2203	
	TRP201				GLN846						THR2207	
					LEU865						ASN2147	
					LEU864							
					ASN299							
Hydrophobic Interactions	LEU657	3.46	LEU657	3.43	LYS298	3.88	LEU1900	3.75	LEU1900	3.66	LEU1900	3.31
	PHE698	3.83	LEU660	3.72	LEU657	3.84	GLN1937	3.75	GLN1937	3.44	PRO2146	3.63
			PHE694	3.60			PRO1940	3.73	GLN2200	3.98	GLU2196	3.97
			PHE694	3.57			GLN2200	4.00			GLN2200	3.77
			PHE698	3.62								

π –sulfur, π-electron cloud between the Aromatic rings of ligands and lone pair of electron cloud of sulfur atom in the receptors; π -π stacked, π-electron cloud between the Aromatic rings; π -π T-shaped, T shaped π-electron cloud between the Aromatic rings; π –alkyl, π-electron cloud between the Aromatic ring of ligand and alkyl group of ligand; Ӑ, Angstrom.

## Discussion

Despite the availability of various treatment strategies, CRC remains among the top ranked most diagnosed and leading causes of cancer deaths globally (Sung et al., 2021). In order to achieve a reasonable shift in the global burden of CRC, the importance of diagnostic and prognostic biomarkers cannot be overemphasized. In the present study, our differential expression and survival analyses indicated that c-MET/EGFR can be an effective biomarker for CRC progression, immune infiltration, CAF infiltration, and poor prognoses, and thus can serve as attractive targets for exploring the development of chemotherapeutic agents against CRC. We found that c-MET and EGFR expression levels were independent biomarkers for clinical prognosis, and their high expressions were correlated with tumor progression and a poor prognosis of CRC. Abnormal expressions of c-MET and EGFR were reported in various human cancers where they support the proliferation and survival of cancer cells (Nicholson et al., 1990; Normanno et al., 2006; Zhang et al., 2018; Chakraborty et al., 2019; Fujiwara et al., 2020; Lee et al., 2020). c-MET and EGFR regulate the synthesis and secretion of several angiogenic growth factors, including basic fibroblast growth factor (bFGF), vascular endothelial growth factor (VEGF), and interleukin (IL)-8, in tumor cells (De Luca et al., 2008). Taken together, these findings suggest that the c-MET/EGFR system is an important mediator within the tumor microenvironment (TME) that results in enhanced tumor growth.

Furthermore, we explored TCGA clinical database to evaluate the frequency and prognostic consequences of c-MET/EGFR genetic alterations in CRC. We found that 3.0 and 4% of the cohorts that respectively harbored genetically altered EGFR and MET had poor prognoses and shorter survival times compared to cohorts without c-MET/EGFR alterations. Genetic alterations of c-MET and EGFR were previously identified and implicated in the progression of several cancers (Bastien et al., 2015; Piccirillo et al., 2015; Yamaura et al., 2020) and were also implicated in the resistance of most cancers to any generation of EGFR-TKIs including the latest third-generation series (Paez et al., 2004; Pao et al., 2005; Guo et al., 2020a). Of importance, we analyzed concurrent gene alterations with c-MET/EGFR genetic alterations, and found that EGFR alteration co-occurred with genetic alterations of *SMARCD2*, *TRIM7*, *MCMDC2*, *HNRNPUL1*, *BEST3*, *GRB10*, *PDE6C*, and *SNX19*, while alterations of *SLC22A2*, *DST*, *AKT3*, and *TAB2* co-occurred with c-MET alterations in CRC. Genetic alterations and concurrent alterations of c-MET/EGFR with other genetic alterations have been well reported in lung cancer (Stuart and Sellers, 2009; Tang et al., 2018; Guo et al., 2020b; Gini et al., 2020), but were underreported in other solid cancers especially CRC, and hence our study is significant in this regard. A recent study reported concurrent alterations of p53 (60–65%), RTKs (5–10%), PIK3CA/KRAS (3–23%), Wnt (5–10%), and cell cycle pathways MYC (7–25%) and NKX2-1 (10–15%) in EGFR-altered cohorts (Gini et al., 2020). Another more-recent study also identified concurrent genetic alterations of *EGFE* with *MEK*, *ALK*, *KRAS*, *ROS1*, *TP53*, *PIK3CA*, and *PTEN* in NSCLC (Guo et al., 2020b), while Tang et al. (Tang et al., 2018) reported coexistent genetic alterations involving *ALK*, *RET*, *ROS1*, and *MET* in 15 cases of lung adenocarcinoma (Tang et al., 2018). In addition to concurrent gene alterations in CRC patients, we found that the genetic alteration of EGFR was associated with overexpression of mRNA levels of TRIM7, BARX2, CFAP43, TPBG, HPSE, GNB5, MARK3, FUT8-AS1, SS18 and KCNK1, while genetic alteration of MET was associated with the overexpression of mRNA levels of *SMAD4*, *MBP*, *IER3IP1*, *OXCT1*, *ALKBH5*, *C18ORF25*, *WDR76*, *TYMS*, *LINC00909*, and *GPR3* in CRC cohorts. We also found that these overexpressed mRNAs were enriched in several KEGG pathways including cell adherent junctions, central carbon metabolism, and EGFR TKI resistance, indicating a potential functional role in driving resistance to therapy. We explored the PPI network and pathways of these mRNAs from a different perspective, and we concluded that multiple components and multiple pathways led to the development and progression of CRC. However, we found that c-MET, ERBB2, and EGFR signaling pathways, MAPKK activation, and negative regulation of cellular responses to drugs appeared to play important roles in the development of CRC. Our findings are therefore relevant in predicting and classifying evolutionary trends of genetic alterations during therapy and could guide the selection of combination therapies appropriate for patients with resistance to TKIs in CRC.

The TME is a complex ecological niche that plays important roles in tumor growth and progression. The TME consists of several components including tumor cells and adjacent non-tumor cells, such as infiltrating immune cells, fibroblasts, signaling molecules, and the extracellular matrix (ECM) (Azizi et al., 2018; Pearce et al., 2018; Zou et al., 2020). Tumor-infiltrating immune cells are well associated with angiogenesis, tumorigenesis, and metastasis of tumor cells, which can in turn regulate the quantity and subsets of immune cells (Taddei et al., 2013). Oncogenic signaling molecules are known to be drivers of immune infiltration and immunotherapeutic responses (Lawal et al., 2021a). Consequently, the present study revealed associations of c-MET/EGFR expressions with infiltration of various immune cells in CRC and predicted poor survival of the cohorts. We also found that both genetic and epigenetic alterations of c-MET/EGFR were associated with immune infiltration, dysfunctional T-cell phenotypes, and poor prognoses of CRC patients, hence adding another layer of prognostic relevance of c-MET/EGFR in CRC.

CAFs are apoptotic-resistant fibroblasts that promote tumorigenic and metastatic properties by secreting cytokines and remodeling the ECM (Erez et al., 2010; Giannoni et al., 2010). We found that associations between c-MET/EGFR expression profiles and CAF infiltration resulted in low cumulative survival of CRC cohorts. These findings could be attributed to the fact that oncogenes induce the CAF phenotype (Lisanti et al., 2013), which in turn inhibits T-cell expansion by mediating the hyper-expression of immune checkpoint molecules in the TME, consequently leading to T-cell allergies (Harryvan et al., 2019), immune therapy failure, aggressive tumor growth, and high death rates in those cohorts. Coherent with our results, a previous study reported that CAFs promote EGFR-TKI resistance via c-MET/IGF-1/ANXA2 signaling in NSCLC (Yi et al., 2018). Another study also reported that CAFs regulate the plasticity of tumor-initiating cells in liver cancer through c-Met/FRA1/HEY1 signaling (Lau et al., 2016), while Lau et al. (2017) reported that CAFs promote the metastasis of ovarian cancer via tumor necrosis factor (TNF)-α/transforming growth factor (TGF)-α/EGFR signaling. Altogether, our study suggested that c-MET/EGFR play important roles in CAF-mediated tumorigenesis and host immune responses to CRC. It is therefore reasonable to conclude that c-MET/EGFR are important biomarker signatures of tumorigenesis, tumor immune invasion, and poor prognoses, and thus can serve as attractive targets for the development of immune/chemotherapeutic strategies against CRC. In addition, our study may be clinically useful in prognosis assessment and follow-up management of immunotherapy of CRC. Immunotherapies are more effective and promising for some tumor patients than others, and our study therefore suggested that c-MET/EGFR could be an important contributor to those less sensitive to CRC immunotherapy compared to other cancer types as reported in a previous study (Banerjea et al., 2005), hence providing a rationale for a combination of c-MET/EGFR antagonists and immunotherapy in CRC. From another perspective, since EGFR aberrations co-occurred with other genetic assaults, developing a potent c-MET/EGFR inhibitor with multi-target potential capable of inhibiting multiple pathways with minimal side effects may open up new windows for long-lasting, multilayer control of CRC. Hence we evaluated NSC777205 and NSC777207, gefitinib-inspired small molecules derivatives of niclosamine, for that purpose.

On the basis of PK studies during the early stages of drug discovery possibly reducing attrition rates (Kola and Landis, 2004), we evaluated NSC777205 and NSC777207 for *in silico* drug-likeness and ADME profiles, and found that both NSC777205 and NSC777207 possessed good drug-likeness properties and obeyed Lipinski’s rule-of-five, a physicochemical-based rule set to evaluate drug candidates for suitability as oral remedy (Pollastri, 2010). The number of rotatable bonds is important in the stereoselectivity of drug molecules for optimal binding with target receptor molecules, while the topological polar surface area (TPSA) and molar refractivity are important for a drug’s transport and biodistribution (Veber et al., 2002; Prasanna and Doerksen, 2009). Values of the TPSA, molar refractivity, and other PK parameters observed for NSC777205 and NSC777207 were within the permissible range of a good drug molecule.

The overall analysis of drug-likeness studies strongly suggested that NSC777205 and NSC777207 possessed good drug-likeness properties for optimal drug transport, bioavailability, intestinal availability, membrane permeability, and eventual interactions with receptor molecules and bioactivity. Interestingly both NSC777205 and NSC777207 also demonstrated *in silico* cytotoxic activities against colon cancer, brain, lung, and bone cancer cell lines with NSC777205 demonstrating higher activity (Pa = 0.31–0.658) than NSC777207 (Pa = 0.303–0.565). BBB penetration is an important limiting factor in the use of chemotherapy for treating glioblastomas and other CNS-associated diseases (Moretti et al., 2015). Interestingly, our *in silico* study suggested that both NSC777205 and NSC777207 had good BBB permeation ability, with NSC777205 demonstrating about 2-fold higher ability than NSC777207. Notably, the synthetic accessibilities of these compounds were lower than 3.5, suggesting that they can relatively easily be synthesized. The overall analysis indicated that NSC777205 demonstrated more-favorable drug likeness, ADMET, and cytotoxic properties than NSC777207, thus suggesting better therapeutic prospects of the former than the latter. Considering these promising PK, ADMET, and biological properties, we synthesized these compound by adapting and optimizing our previously established protocols (Chen et al., 2014) and evaluated their anticancer activities against the NCI-60 human tumor cell lines.

To our delight, the *in vitro* anticancer study revealed that both NSC777205 and NSC777207 exhibited wide spectra of antiproliferative activities with GI_50_ values of <6 µM against NCI-60 cell line panels with selective cytotoxic preferences for melanoma, renal, CNS, colon, and NSCLC cell lines, suggesting that the compounds were not generally toxic to growing cells but were selective for melanoma, renal, CNS, colon, and NSCLC cells. This observation corroborates with the *in silico* structure-activity-related cytotoxic predictions for NSC777205 and NSC777207 against cancer cell lines, which **i**ndicated that both compounds exhibited cytotoxic activities against colon (HCT-116 and RKO) brain (Hs683), lung (A549) and bone cancer (SJSA-1) cell lines with NSC777205 demonstrating higher activity (Pa = 0.31–0.658) than NSC777207 (Pa = 0.303–0.565). Of importance, among all cell lines, colon cancer cell lines stood out uniquely with all seven cell lines demonstrating strict antiproliferative sensitivity and no cytotoxic response to single-dose (10 μM) treatment of either NSC777205 or NSC777207. This suggests that NSC777205 and NSC777207 would be very useful in growth inhibition and treatment of CRC. Tumors often develop resistance to a wide range of anticancer drugs. Therefore, drug resistance represents an important problem in cancer chemotherapy (Baguley, 2010; Alfarouk et al., 2015). Intriguingly, NSC777205 and NSC777207 demonstrated higher anticancer activities on multidrug resistant NC1-60 human tumor cell lines including the SK-OV-3, OVCAR-3 OVCAR-4, and NCI/ADR-RES cell lines than the corresponding naïve cell lines of the same origin (OVCAR-8 and IGROV1). These cell lines have been well profiled and established to be resistant to multiple clinical drugs (Louie et al., 1985; Lorenzi et al., 2009). It is therefore plausible that since NSC777205 and NSC777207 demonstrated promising activity against these cell lines, they may be useful in treating multidrug-resistant cancers. Furthermore, among the NCI-60 cell lines investigated in this study, the leukemia RPMI-8226 and melanoma SK-MEL-28 cell lines were identified to harbor EGFR mutations which led to their resistance to 12 TKIs, including erlotinib (Ikediobi et al., 2006; Liu et al., 2007). It is therefore noteworthy that both NSC777205 and NSC777207 exhibited high antiproliferative activity against these cell lines, hence suggesting their applicability in treating TKI-resistant tumors. In line with the *in silico* cytotoxic activities and ADMET properties, our *in vitro* anticancer study also indicated that NSC777205 had higher anticancer activities than NSC777207 against the nine cancer types evaluated. These higher activities of NSC777205 could therefore be attributed to its estimated higher BBB permeation and favorable ADME and physicochemical properties that give it better stability and a greater ability to interact with targets than NSC777207. Hence, we evaluated the possible mechanistic properties of these compounds via an *in silico* study and molecular docking simulations of ligand-receptor interactions.

Identification of molecular targets is an important aspect of developing novel small molecules for chemotherapeutic purposes, and a number of computational tools that have been developed and widely employed for these purposes have had translational success in preclinical and clinical practice. However, due to differences in embedded principles for target identification by these tools and also the vast diversity of targets, it is recommended to use multiple computational tools and identify common targets predicted for certain drug candidates (frontiers). In compliance with this recommendation, we used four computational tools including the COMPARE algorithm developed by the USA NCI to identify the most probable targets for NSC7772015 and NSC777207. In the COMPARE analysis, the anticancer fingerprints of NSC777205 and NSC777207 corresponded to anticancer fingerprints of known inhibitors of c-MET/EGFR/mTOR/PI3K pathways. Furthermore, the most highly correlated NCI synthetic compound (*p* = 0.78) was 3-(2-fluoro-4-iodophenyl)-7-methoxy-2H-benzo[e](Fidler et al., 2016; Sung et al., 2021) oxazine-2,4(3H)-dione, whose detailed mechanistic targets were reported to be PI3K, AKT, mTOR, MEK, and EGFR (Lawal et al., 2021c). In addition, N-(2,4-difluorophenyl)-2', 4'-difluoro-4-hydroxybiphenyl-3-carboxamide and (4-(6-(2,4-difluorophenyl)-2,4-dioxo-2H-benzo[e] (Fidler et al., 2016; Sung et al., 2021)-oxazin-3(4H)-yl) benzonitrile which also showed significant correlations with anticancer fingerprints of NSC777205 and NSC777207 were mechanistically reported, and signal transducer and activator of transcription 3 (STAT3)/cyclin-dependent kinases (CDKs)/PIK3CB/EGFR were identified among other targets (Lawal et al., 2021b). c-MET phosphorylates and triggers intracellular signaling cascades that lead to activation of key molecules such as the extracellular signal-regulated kinase 1/2 (ERK1/2), PI3K/AKT signaling, and STAT3 (Organ and Tsao, 2011; Wood et al., 2021). It is therefore plausible that the COMPARE analysis inferred that both NSC777205 and NSC777207 may also alter the expression capacity of STAT3 in addition to the promising mechanistic target of the c-MET/EGFR/PI3K/mTOR pathways. Collectively, we found that c-MET/EGFR/P13K/mTOR satisfied the target criteria set by the four tools we used. These tools hence suggested that c-MET/EGFR/P13K/mTOR are the most probable targets and mechanisms of action of NSC7772015 and NSC7772017.

Molecular docking aids the identification of drug lead molecules by unraveling possible interactions between a drug candidate and protein targets, providing structure-activity relationships and therapeutic mechanisms of drug candidates prior to *in vitro* or *in vivo* validation. Non-covalent interactions such as hydrogen bonds, hydrophobic, ionic, and π-interactions, and van der Waals forces occurring between ligands and receptors are important for stabilization of a ligand within the binding cavity of a receptor (Zhao and Huang, 2011). Our docking analysis revealed that NSC777205 and NSC777207 docked well into the binding cavities of c-MET, EGFR, mTOR, and P13K by various non-covalent interactions and with binding affinities ranging -6.8∼-8.9 kcal/mol. These collective interactions would lead to strong stabilization of NSC777205 and NSC777207 in receptor cavities, which could lead to competitive or non-competitive alterations in the normal expressions and functional properties of the proteins, thus compromising the sustenance and survival of the oncogenic pathways that depend on the proteins for their hyperactivity. Hydrophobic interactions play important roles in the formation of ligand-receptor complexes. Although NSC777205 and NSC777207 shared common interacting residues with the receptors, in most cases, NSC777205 demonstrated more-robust and a higher number of hydrogen bonds, hydrophobic contacts, and van der Waals forces with the receptors than did NSC777207. These higher hydrophobic interactions would enhance the stabilization of the energetically favored NSC777205 in an open conformation surrounding the receptors and further increase its biological activity (Patil et al., 2010; Lawal et al., 2021c). In addition, the higher van der Waals forces would create a strong cohesive environment, that further stabilizes the complex (Arthur and Uzairu, 2019). Both NSC777205 and NSC777207 demonstrated similar tendencies for inhibiting c-MET and EGFR as revealed by the most common interacting residues and similar binding affinities to the binding sites of c-MET and EGFR; however, NSC777205 had higher hydrophobic contacts, H-bonds, halogens, and π-interactions with EGFR than did NSC777207. In addition, the higher number of H-bonds and van der Waals forces around the ligand backbone could be responsible for the higher affinity that NSC777205 had for PI3K than did NSC777207.

Both NSC777205 and NSC777207 exhibited high similarity with gefitinib and crizotinib, known clinical EGFR and c-MET inhibitors, respectively, in terms of common interacting residues with EGFR and c-MET and similar binding affinities with EGFR, suggesting that both NSC777205 and NSC777207 could be as promising as gefitinib and crizotinib at inhibiting EGFR and c-MET, serving as potential dual targets and thus superseding the strength of individual clinical drugs in arresting cancer proliferation and aggressiveness for which EGFR and c-MET are implicated. This multi-target potency of NSC777205 and NSC777207 could be attributed to the observed activities of NSC777205 and NSC777207 against NCI cell lines that are known to be multi-drug resistant including those that are resistant to more than 10 panels of TKIs (Liu et al., 2007).

Collectively, our study revealed that structural hybridization strategies yielded novel and potential c-MET/EGFR/PI3K/mTOR multi-target compounds, NSC777205 and NSC777205, with promising prospects for treating melanoma, renal, CNS, colon, and NSCLC cell lines. Although NSC777205 and NSC777207 share common interacting residues with the receptors, the overall structure-activity relationship study revealed that the addition of an –OCH_3_ group to the salicylic core of NSC777207 was not favorable, as the added moiety did not contribute significant interactions with the receptor-binding residues and also led to overall less-favorable ADMET, physicochemical, and drug-likeness properties and weaker anticancer activities against the panel of NCI cell lines compared to the properties and activities demonstrated by NSC777205 that has no –OCH_3_ substituent group. However, further *in vitro* and *in vivo* analyses in tumor-bearing mice are ongoing in our lab to support this claim and to unravel the full therapeutic efficacies of NSC777205 and 777207 in CRC.

## Conclusion

In conclusion, our results indicated that, c-Met/EGFR are important biomarker signatures of cancer-associated fibroblasts and tumor immune infiltration, and are of clinical relevance in colorectal cancer. Genetic and epigenetic alterations of c-MET/EGFR were associated with dysfunctional T-cell phenotypes, and poor prognoses of the cohorts. c-MET/EGFR/PI3K/mTOR are therapeutic targets for NSC777205 and NSC777207 with consequent selective cytotoxic preferences for melanoma, renal, CNS, colon, and NSCLC cell lines. However, the removal of an –OCH_3_ substituent group from the salicylic core of NSC777205 was responsible for more highly favorable drug-likeness properties and anticancer activities, and more-robust interactions with c-MET/EGFR/PI3K/mTOR than those of NSC777207.

## Data Availability

The raw data supporting the conclusions of this article will be made available by the authors, without undue reservation, to any qualified researcher.
