# Supplementary material for: Pro-Oncogenic c-Met/EGFR, Biomarker Signatures of the Tumor Microenvironment are Clinical and Therapy Response Prognosticators in Colorectal Cancer, and Therapeutic Targets of 3-Phenyl-2H-benzo[e][1,3]-Oxazine-2,4(3H)-Dione Derivatives
Source: Front Pharmacol. 2021 Aug 27;12:691234. doi: 10.3389/fphar.2021.691234 (PMC8429938; doi:10.3389/fphar.2021.691234)
Supplement: Supplementary file 1 [file DataSheet1.docx]

**Supplementary table 1:** c-MET/EGFR expressions are associated with tumor immune infiltrations of colorectal cancer cohorts

| EGFR | | | |  | c-MET | | | |
| --- | --- | --- | --- | --- | --- | --- | --- | --- |
| cancer | variable | partial.cor | p |  | cancer | variable | partial.cor | p |
| COAD | Purity | -0.0716 | 0.149309 |  | COAD | Purity | 0.01029 | 0.836045 |
| COAD | B Cell | 0.173612 | 0.000456 |  | COAD | B Cell | 0.134019 | 0.006985 |
| COAD | CD8+ T Cell | 0.172416 | 0.000484 |  | COAD | CD8+ T Cell | 0.152658 | 0.002038 |
| COAD | CD4+ T Cell | 0.537333 | 1.88E-31 |  | COAD | CD4+ T Cell | 0.259385 | 1.33E-07 |
| COAD | Macrophage | 0.348739 | 5.37E-13 |  | COAD | Macrophage | 0.212569 | 1.64E-05 |
| COAD | Neutrophil | 0.298804 | 1.03E-09 |  | COAD | Neutrophil | 0.185771 | 0.000183 |
| COAD | Dendritic Cell | 0.355964 | 1.88E-13 |  | COAD | Dendritic Cell | 0.201488 | 4.72E-05 |
| READ | Purity | -0.18717 | 0.026802 |  | READ | Purity | 0.017459 | 0.83777 |
| READ | B Cell | 0.374096 | 5.72E-06 |  | READ | B Cell | 0.18019 | 0.033784 |
| READ | CD8+ T Cell | 0.286481 | 0.000628 |  | READ | CD8+ T Cell | 0.32561 | 9.18E-05 |
| READ | CD4+ T Cell | 0.293164 | 0.000461 |  | READ | CD4+ T Cell | 0.048388 | 0.571623 |
| READ | Macrophage | 0.386049 | 2.68E-06 |  | READ | Macrophage | 0.269044 | 0.001362 |
| READ | Neutrophil | 0.191902 | 0.024143 |  | READ | Neutrophil | 0.283537 | 0.000752 |
| READ | Dendritic Cell | 0.323022 | 0.000105 |  | READ | Dendritic Cell | 0.090401 | 0.289894 |

**Supplementary table 2:** Microbiome Signature Over Expressed In Colorectal Cancer Cohorts With Altered EGFR

| **Entity Name** | **Altered group** | **Unaltered group** | **Altered group** | **Unaltered group** | **Log Ratio** | **p-Value** | **q-Value** | **Higher in** |
| --- | --- | --- | --- | --- | --- | --- | --- | --- |
| Aquamavirus | 1.6 | 2.07 | 0.29 | 0.96 | -0.47 | 1.82E-05 | 0.0239 | Unaltered group |
| Actinosynnema | -1.31 | -0.89 | 0.29 | 0.87 | -0.42 | 5.63E-05 | 0.0239 | Unaltered group |
| Castellaniella | 0.97 | 1.59 | 0.44 | 0.9 | -0.61 | 8.47E-05 | 0.0239 | Unaltered group |
| Moorella | 1.33 | 1.76 | 0.31 | 0.83 | -0.43 | 9.18E-05 | 0.0239 | Unaltered group |
| Betaretrovirus | 4.06 | 4.47 | 0.29 | 0.94 | -0.41 | 9.45E-05 | 0.0239 | Unaltered group |
| Pimelobacter | 1.19 | 1.64 | 0.34 | 0.79 | -0.45 | 1.25E-04 | 0.0239 | Unaltered group |
| Arcanobacterium | 2.2 | 2.79 | 0.44 | 1.05 | -0.58 | 1.33E-04 | 0.0239 | Unaltered group |
| Cryobacterium | -0.83 | -0.29 | 0.4 | 0.87 | -0.53 | 1.36E-04 | 0.0239 | Unaltered group |

**Table 3:** c-MET/EGFR genetic alteration co-occurrence with other genes alterations in colorectal cancer

| **Gene** | **Cytoband** | **Altered group** | **Unaltered group** | **Log Ratio** | **p-Value** | **q-Value** | **Enriched in** |
| --- | --- | --- | --- | --- | --- | --- | --- |
|  | **EGFR** | | | | | | |
| SMARCD2 | 17q23.3 | 6 (21.43%) | 2 (0.34%) | 5.98 | 1.30E-07 | 2.33E-03 | Altered group |
| TRIM7 | 5q35.3 | 5 (17.86%) | 2 (0.34%) | 5.72 | 2.60E-06 | 0.0233 | Altered group |
| MCMDC2 | 8q13.1 | 5 (17.86%) | 3 (0.51%) | 5.14 | 6.72E-06 | 0.0401 | Altered group |
| HNRNPUL1 | 19q13.2 | 9 (32.14%) | 28 (4.74%) | 2.76 | 1.10E-05 | 0.0401 | Altered group |
| BEST3 | 12q15 | 6 (21.43%) | 8 (1.35%) | 3.98 | 1.16E-05 | 0.0401 | Altered group |
| GRB10 | 7p12.1 | 7 (25.00%) | 14 (2.37%) | 3.4 | 1.34E-05 | 0.0401 | Altered group |
| PDE6C | 10q23.33 | 6 (21.43%) | 9 (1.52%) | 3.81 | 1.87E-05 | 0.0418 | Altered group |
| SNX19 | 11q24.3-q25 | 6 (21.43%) | 9 (1.52%) | 3.81 | 1.87E-05 | 0.0418 | Altered group |
| **c-MET** | | | | | | | |
| SLC22A2 | 6q25.3 | 6 (37.50%) | 12 (1.99%) | 4.24 | 1.65E-06 | 0.0217 | Altered group |
| DST | 6p12.1 | 9 (56.25%) | 50 (8.29%) | 2.76 | 2.42E-06 | 0.0217 | Altered group |
| AKT3 | 1q43-q44 | 5 (31.25%) | 8 (1.33%) | 4.56 | 6.69E-06 | 0.0399 | Altered group |
| TAB2 | 6q25.1 | 5 (31.25%) | 9 (1.49%) | 4.39 | 1.02E-05 | 0.0459 | Altered group |

**Supplementary Table 4:** Over-expressed mRNA in c-MET/EGFR altered colorectal cancer cohorts

| **Gene** | **Cytoband** | **Altered group** | **Unaltered group** | **Altered group** | **Unaltered group** | **Log Ratio** | **p-Value** | **q-Value** | **Higher expression in** |
| --- | --- | --- | --- | --- | --- | --- | --- | --- | --- |
| EGFR | | | | | | | | | |
| TRIM7 | 5q35.3 | 7.69 | 5.43 | 1.43 | 2.04 | 2.26 | 2.97E-06 | 3.32E-04 | Altered group |
| BARX2 | 11q24.3 | 7.39 | 5.82 | 1.05 | 1.96 | 1.57 | 5.68E-06 | 5.93E-04 | Altered group |
| CFAP43 | 10q25.1 | 3.67 | 2.42 | 0.84 | 1.16 | 1.25 | 7.05E-06 | 7.06E-04 | Altered group |
| TPBG | 6q14.1 | 9.25 | 8.06 | 0.85 | 1.15 | 1.19 | 1.41E-05 | 1.26E-03 | Altered group |
| HPSE | 4q21.23 | 7.85 | 6.58 | 0.93 | 1.05 | 1.27 | 2.07E-05 | 1.75E-03 | Altered group |
| GNB5 | 15q21.2 | 8.28 | 7.61 | 0.5 | 0.84 | 0.68 | 2.38E-05 | 1.93E-03 | Altered group |
| MARK3 | 14q32.32-q32.33 | 10.17 | 9.87 | 0.23 | 0.37 | 0.3 | 4.61E-05 | 3.43E-03 | Altered group |
| FUT8-AS1 | 14q23.3 | 5.34 | 4.33 | 0.81 | 1.09 | 1.01 | 5.90E-05 | 4.24E-03 | Altered group |
| SS18 | 18q11.2 | 10.31 | 9.78 | 0.43 | 0.55 | 0.53 | 6.94E-05 | 4.82E-03 | Altered group |
| KCNK1 | 1q42.2 | 9.7 | 8.83 | 0.71 | 1.24 | 0.87 | 7.47E-05 | 5.08E-03 | Altered group |
| **c-MET** | | | | | | | | | |
| SMAD4 | 18q21.2 | 10.34 | 9.89 | 0.26 | 0.67 | 0.45 | 3.29E-08 | 9.26E-06 | Altered group |
| MBP | 18q23 | 10.16 | 9.68 | 0.29 | 0.63 | 0.48 | 7.22E-08 | 1.83E-05 | Altered group |
| IER3IP1 | 18q21.1 | 10.22 | 9.7 | 0.34 | 0.54 | 0.52 | 2.58E-07 | 5.61E-05 | Altered group |
| OXCT1 | 5p13.1 | 9.7 | 8.66 | 0.7 | 1.25 | 1.04 | 5.10E-07 | 9.92E-05 | Altered group |
| ALKBH5 | 17p11.2\|17p11.2 | 10.93 | 10.51 | 0.3 | 0.43 | 0.43 | 8.41E-07 | 1.48E-04 | Altered group |
| C18ORF25 | 18q21.1 | 9.09 | 8.6 | 0.35 | 0.51 | 0.49 | 1.39E-06 | 2.29E-04 | Altered group |
| WDR76 | 15q15.3 | 8.61 | 7.84 | 0.57 | 0.73 | 0.77 | 2.18E-06 | 3.21E-04 | Altered group |
| TYMS | 18p11.32 | 10.52 | 9.66 | 0.63 | 0.82 | 0.85 | 2.56E-06 | 3.66E-04 | Altered group |
| LINC00909 | 18q22.3 | 7.24 | 6.87 | 0.28 | 0.58 | 0.37 | 2.84E-06 | 3.92E-04 | Altered group |
| GPR3 | 1p36.11 | 5.95 | 4.74 | 0.92 | 1.03 | 1.21 | 3.92E-06 | 5.24E-04 | Altered group |

**Supplementary Table 5:** PharmMapper Prediction of NSC777205 and NSC777207 targets

|  | NSC777207 (PharmMapper Prediction) | | | | |  |
| --- | --- | --- | --- | --- | --- | --- |
| Pharma Model | Num Feature | Norm Fit | zscore | Num Hydrophobic | Name | Uniplot |
| 1m66_v | 3 | 0.9813 | 1.11829 | 3 | Glycerol-3-phosphate dehydrogenase [NAD+], glycosomal | P90551 |
| 1x81_v | 3 | 0.9806 | 1.16063 | 3 | Protein farnesyltransferase/geranylgeranyltransferase type-1 subunit alpha | Q04631 |
| 1uki_v | 3 | 0.9756 | 1.01229 | 2 | Mitogen-activated protein kinase 8 | MK08_HUMAN |
| 1pmv_v | 3 | 0.9695 | 0.891003 | 2 | Mitogen-activated protein kinase 10 | MK10_HUMAN |
| 1pme_v | 3 | 0.9389 | 0.647472 | 3 | Mitogen-activated protein kinase 1 | MK01_HUMAN |
| 2o65_v | 3 | 0.9151 | 0.373572 | 3 | Proto-oncogene serine/threonine-protein kinase Pim-1 | PIM1_HUMAN |
| 2p3g_v | 3 | 0.8304 | -0.67468 | 3 | MAP kinase-activated protein kinase 2 | P49137 |
| 1nwe_v | 5 | 0.7664 | 3.74477 | 2 | Tyrosine-protein phosphatase non-receptor type 1 | PTN1_HUMAN |
| 1zyj_v | 4 | 0.7395 | 0.878389 | 3 | Mitogen-activated protein kinase 14 | Q16539 |
| 1agw_v | 4 | 0.7384 | 0.994274 | 2 | Basic fibroblast growth factor receptor 1 | FGFR1_HUMAN |
| 2vtn_v | 4 | 0.7321 | 0.963471 | 2 | Cell division protein kinase 2 | P24941 |
| 1o4f_v | 4 | 0.723 | 0.467844 | 1 | Proto-oncogene tyrosine-protein kinase Src | SRC_HUMAN |
| 2pe0_v | 4 | 0.7204 | 0.802733 | 3 | 3-phosphoinositide-dependent protein kinase 1 | PDPK1_HUMAN |
| 2ywp_v | 4 | 0.7143 | 0.869014 | 2 | Serine/threonine-protein kinase Chk1 | CHK1_HUMAN |
| 1m17_v | 4 | 0.6986 | 0.548975 | 2 | Epidermal growth factor receptor | EGFR_HUMAN |
| 2rl5_v | 5 | 0.6487 | 1.7071 | 4 | Vascular endothelial growth factor receptor 2 | P35968 |
| 2rfn_v | 5 | 0.5884 | 0.785022 | 4 | Hepatocyte growth factor receptor | P08581 |
| 1m9t_v | 4 | 0.5838 | -1.76711 | 3 | Nitric oxide synthase, inducible | P29477 |
| 1m7w_v | 5 | 0.5783 | 0.035044 | 4 | Hepatocyte nuclear factor 4-alpha | P22449 |
| 2vnt_v | 5 | 0.5626 | 0.545182 | 1 | Urokinase-type plasminogen activator | UROK_HUMAN |
| 1oec_v | 5 | 0.5417 | -0.32385 | 3 | Fibroblast growth factor receptor 2 | P21802 |
| 1dm7_v | 5 | 0.5101 | -0.28784 | 1 | Nitric oxide synthase, endothelial | P29473 |
|  |  |  |  |  |  |  |
|  | NSC777205 (PharmMapper Prediction) | | | | | |
| Pharma Model | Num Feature | Norm Fit | zscore | Num Hydrophobic | Name | Uniplot |
| 1uki_v | 3 | 0.9583 | 0.839877 | 2 | Mitogen-activated protein kinase 8 | MK08_HUMAN |
| 1pmv_v | 3 | 0.9558 | 0.709323 | 2 | Mitogen-activated protein kinase 10 | MK10_HUMAN |
| 1pme_v | 3 | 0.9239 | 0.496708 | 3 | Mitogen-activated protein kinase 1 | MK01_HUMAN |
| 2o65_v | 3 | 0.9151 | 0.389832 | 3 | Proto-oncogene serine/threonine-protein kinase Pim-1 | PIM1_HUMAN |
| 2p3g_v | 3 | 0.8304 | -0.66088 | 3 | MAP kinase-activated protein kinase 2 | P49137 |
| 1agw_v | 4 | 0.7364 | 0.985027 | 2 | Basic fibroblast growth factor receptor 1 | FGFR1_HUMAN |
| 1zyj_v | 4 | 0.7314 | 0.761317 | 3 | Mitogen-activated protein kinase 14 | Q16539 |
| 1m17_v | 4 | 0.6981 | 0.557271 | 2 | Epidermal growth factor receptor | EGFR_HUMAN |
| 2ywp_v | 4 | 0.6797 | 0.416247 | 2 | Serine/threonine-protein kinase Chk1 | CHK1_HUMAN |
| 1vjy_v | 4 | 0.6617 | -0.36276 | 3 | TGF-beta receptor type-1 | TGFR1_HUMAN |
| 2rl5_v | 5 | 0.6488 | 1.73203 | 4 | Vascular endothelial growth factor receptor 2 | P35968 |
| 2rfn_v | 5 | 0.5876 | 0.788402 | 4 | Hepatocyte growth factor receptor | P08581 |
| 2w1g_v | 5 | 0.5244 | -0.1256 | 2 | Serine/threonine-protein kinase 6 | O14965 |
| 1oec_v | 5 | 0.5148 | -0.88036 | 3 | Fibroblast growth factor receptor 2 | P21802 |
| 3bbt_v | 6 | 0.4907 | 0.452106 | 5 | Receptor tyrosine-protein kinase erbB-4 | ERBB4_HUMAN |
| 2f57_v | 5 | 0.43 | -1.64541 | 3 | Serine/threonine-protein kinase PAK 7 | PAK7_HUMAN |
| 1lv2_v | 7 | 0.4226 | -0.01695 | 5 | Hepatocyte nuclear factor 4-gamma | Q14541 |
| 1tfg_v | 9 | 0.4202 | 2.09003 | 4 | Transforming growth factor beta-2 | TGFB2_HUMAN |
| 1xjd_v | 7 | 0.4141 | 0.023532 | 4 | Protein kinase C theta type | KPCT_HUMAN |
| 1jk7_v | 9 | 0.3324 | 0.117554 | 6 | Serine/threonine-protein phosphatase PP1-gamma catalytic subunit | P36873 |
| 1x0n_v | 9 | 0.3256 | -0.11646 | 5 | Growth factor receptor-bound protein 2 | GRB2_HUMAN |
| 1pic_v | 9 | 0.3021 | -0.45949 | 5 | Phosphatidylinositol 3-kinase regulatory subunit alpha | P85A_HUMAN |
| 1b55_v | 12 | 0.2331 | -0.73492 | 0 | Tyrosine-protein kinase BTK | BTK_HUMAN |
| 1l8j_v | 13 | 0.2261 | -0.19507 | 11 | Endothelial protein C receptor | EPCR_HUMAN |

**Supplementary Table 6:** PASS prediction of NSC777205 and NSC777207 targets

| Pa | Pi | NSC777205 Target |  | Pa | Pi | NSC777207 Targets |
| --- | --- | --- | --- | --- | --- | --- |
| 0,559 | 0,026 | Analgesic |  | 0,427 | 0,083 | Antineoplastic (non-Hodgkin's lymphoma) |
| 0,582 | 0,078 | Antineurotic |  | 0,425 | 0,087 | HIF1A expression inhibitor |
| 0,464 | 0,004 | CNS active muscle relaxant |  | 0,331 | 0,018 | Hepatocyte growth factor antagonist |
| 0,467 | 0,033 | Analgesic, non-opioid |  | 0,324 | 0,016 | CNS active muscle relaxant |
| 0,465 | 0,054 | Antineoplastic (non-Hodgkin's lymphoma) |  | 0,340 | 0,037 | Endothelial growth factor antagonist |
| 0,375 | 0,012 | Hepatocyte growth factor antagonist |  | 0,326 | 0,040 | Hepatic disorders treatment |
| 0,310 | 0,049 | Endothelial growth factor antagonist |  | 0,364 | 0,086 | Kinase inhibitor |
| 0,336 | 0,077 | Antiinfective |  | 0,369 | 0,112 | Anti-inflammatory |
| 0,348 | 0,124 | Antiinflammatory |  | 0,298 | 0,070 | Antiobesity |
| 0,227 | 0,030 | Renal disease treatment |  | 0,307 | 0,133 | JAK2 expression inhibitor |
| 0,331 | 0,150 | HIF1A expression inhibitor |  | 0,296 | 0,185 | MAP kinase inhibitor |
| 0,286 | 0,147 | JAK2 expression inhibitor |  | 0,139 | 0,038 | VEGF expression inhibitor |
| 0,153 | 0,014 | Inositol 1,4,5-trisphosphate 3-kinase inhibitor |  | 0,223 | 0,137 | Antineoplastic (small cell lung cancer) |
| 0,288 | 0,159 | Kinase inhibitor |  | 0,289 | 0,241 | Inositol 1,4,5-trisphosphate 3-kinase inhibitor |
| 0,232 | 0,130 | MAP kinase kinase 4 inhibitor |  | 0,274 | 0,234 | TP53 expression enhancer |
| 0,156 | 0,071 | HERG channel blocker |  | 0,081 | 0,050 | Multidrug resistance-associated protein inhibitor |
| 0,143 | 0,066 | TH expression enhancer |  | 0,207 | 0,185 | TNF expression inhibitor |
| 0,124 | 0,061 | VEGF expression inhibitor |  | 0,131 | 0,109 | Tumour necrosis factor antagonist |
| 0,242 | 0,220 | EIF4E expression inhibitor |  | 0,074 | 0,071 | Protein kinase B stimulant |

Pa>Pi

**Supplementary Table 7:** Swiss target prediction of NSC777205 and NSC777207 targets

|  | **NSC777205** | | | | |
| --- | --- | --- | --- | --- | --- |
| **Target** | **Common name** | **Uniprot ID** | **ChEMBL ID** | **Target Class** | **Known actives (3D/2D)** |
| Serine/threonine-protein kinase PIM1 | PIM1 | P11309 | CHEMBL2147 | Kinase | 39 / 0 |
| Serine/threonine-protein kinase 33 | STK33 | Q9BYT3 | CHEMBL6005 | Kinase | 13 / 0 |
| Tyrosine-protein kinase ABL | ABL1 | P00519 | CHEMBL1862 | Kinase | 15 / 0 |
| Monoamine oxidase B | MAOB | P27338 | CHEMBL2039 | Oxidoreductase | 71 / 0 |
| Serine/threonine-protein kinase AKT | AKT1 | P31749 | CHEMBL4282 | Kinase | 9 / 0 |
| Cytochrome P450 1A1 | CYP1A1 | P04798 | CHEMBL2231 | Cytochrome P450 | 1 / 0 |
| Epidermal growth factor receptor erbB1 | EGFR | P00533 | CHEMBL203 | Kinase | 128 / 0 |
| PI3-kinase p110-beta subunit | PIK3CB | P42338 | CHEMBL3145 | Enzyme | 10 / 0 |
| PI3-kinase p110-gamma subunit | PIK3CG | P48736 | CHEMBL3267 | Enzyme | 12 / 0 |
| PI3-kinase p110-alpha subunit | PIK3CA | P42336 | CHEMBL4005 | Enzyme | 27 / 0 |
| Vascular endothelial growth factor receptor 1 | FLT1 | P17948 | CHEMBL1868 | Kinase | 17 / 0 |
| Platelet-derived growth factor receptor beta | PDGFRB | P09619 | CHEMBL1913 | Kinase | 7 / 0 |
| Cyclin-dependent kinase 2/cyclin A | CDK2 CCNA1 CCNA2 | P24941 P78396 P20248 | CHEMBL2094128 | Other cytosolic protein | 19 / 0 |
| Serine/threonine-protein kinase mTOR | MTOR | P42345 | CHEMBL2842 | Kinase | 369 / 0 |
| Tyrosine-protein kinase SYK | SYK | P43405 | CHEMBL2599 | Kinase | 14 / 0 |
| Vascular endothelial growth factor receptor 2 | KDR | P35968 | CHEMBL279 | Kinase | 55 / 0 |
| Serine/threonine protein phosphatase PP1-alpha catalytic subunit | PPP1CA | P62136 | CHEMBL2164 | Phosphatase | 2 / 0 |
|  |  |  |  |  |  |
|  | **NSC777207** | | | | |
| **Target** | **Common name** | **Uniprot ID** | **ChEMBL ID** | **Target Class** | **Known actives (3D/2D)** |
| PI3-kinase p110-alpha subunit | PIK3CA | P42336 | CHEMBL4005 | Enzyme | 662 / 0 |
| Nitric oxide synthase, inducible | NOS2 | P35228 | CHEMBL4481 | Enzyme | 101 / 0 |
| Receptor protein-tyrosine kinase erbB-2 | ERBB2 | P04626 | CHEMBL1824 | Kinase | 171 / 0 |
| PI3-kinase p110-delta subunit | PIK3CD | O00329 | CHEMBL3130 | Enzyme | 301 / 0 |
| Serine/threonine-protein kinase TBK1 | TBK1 | Q9UHD2 | CHEMBL5408 | Kinase | 25 / 0 |
| PI3-kinase p110-delta/p85-alpha | PIK3CD PIK3R1 | O00329 P27986 | CHEMBL2111432 | Enzyme | 45 / 0 |
| PI3-kinase p110-beta subunit | PIK3CB | P42338 | CHEMBL3145 | Enzyme | 198 / 0 |
| Serine/threonine-protein kinase Aurora-B | AURKB | Q96GD4 | CHEMBL2185 | Kinase | 210 / 0 |
| Vascular endothelial growth factor receptor 2 | KDR | P35968 | CHEMBL279 | Kinase | 847 / 0 |
| Serine/threonine-protein kinase Aurora-A | AURKA | O14965 | CHEMBL4722 | Kinase | 364 / 0 |
| Serine/threonine-protein kinase mTOR | MTOR | P42345 | CHEMBL2842 | Kinase | 369 / 0 |
| PI3-kinase p110-gamma subunit | PIK3CG | P48736 | CHEMBL3267 | Enzyme | 262 / 0 |
| Protein kinase C (PKC) | PRKCZ | Q05513 | CHEMBL3438 | Kinase | 12 / 0 |
| Vascular endothelial growth factor receptor 1 | FLT1 | P17948 | CHEMBL1868 | Kinase | 128 / 0 |
| TGF-beta receptor type I | TGFBR1 | P36897 | CHEMBL4439 | Kinase | 214 / 0 |
| Mitogen-activated protein kinase kinase kinase 11 | MAP3K11 | Q16584 | CHEMBL2708 | Kinase | 6 / 0 |
| Platelet-derived growth factor receptor beta | PDGFRB | P09619 | CHEMBL1913 | Kinase | 127 / 0 |
| Inhibitor of nuclear factor kappa B kinase beta subunit | IKBKB | O14920 | CHEMBL1991 | Kinase | 96 / 0 |
| Serine/threonine-protein kinase PIM1 | PIM1 | P11309 | CHEMBL2147 | Kinase | 156 / 0 |
| PI4-kinase beta subunit | PI4KB | Q9UBF8 | CHEMBL3268 | Enzyme | 37 / 0 |
| Inhibitor of NF-kappa-B kinase (IKK) | CHUK | O15111 | CHEMBL3476 | Kinase | 27 / 0 |


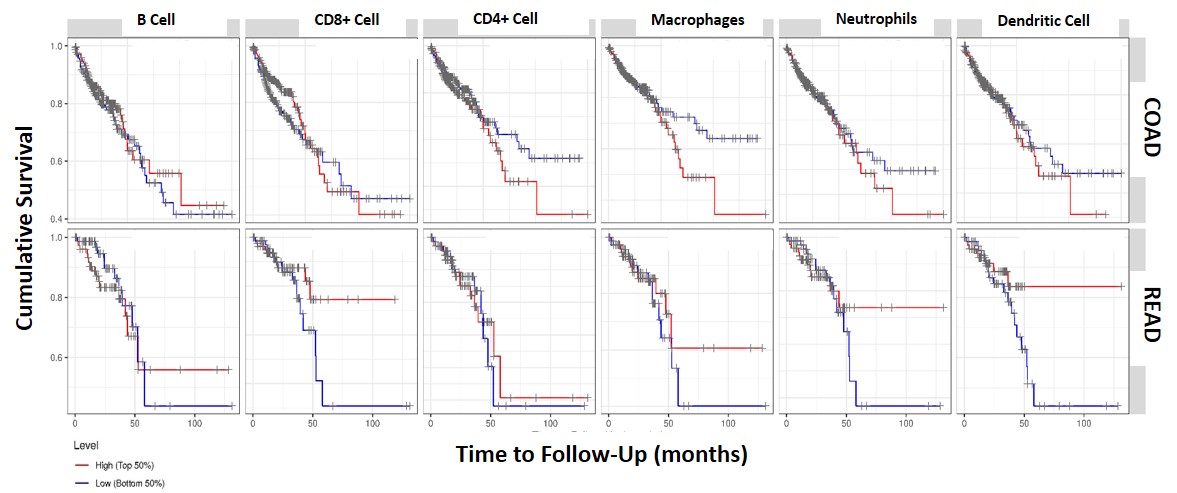
Supplementary Figure 1: Survival analysis of the infiltration of six types of immune cells in COAD and READ. A Kaplan-Meier analysis revealed that infiltration levels of CD4+ T cells, macrophages, neutrophils, and dendritic cells were correlated with poor survival outcomes in COAD, while low infiltration levels of CD8+ T cell, B cells, macrophages, neutrophils, and dendritic cells were correlated with poor survival outcomes in READ.


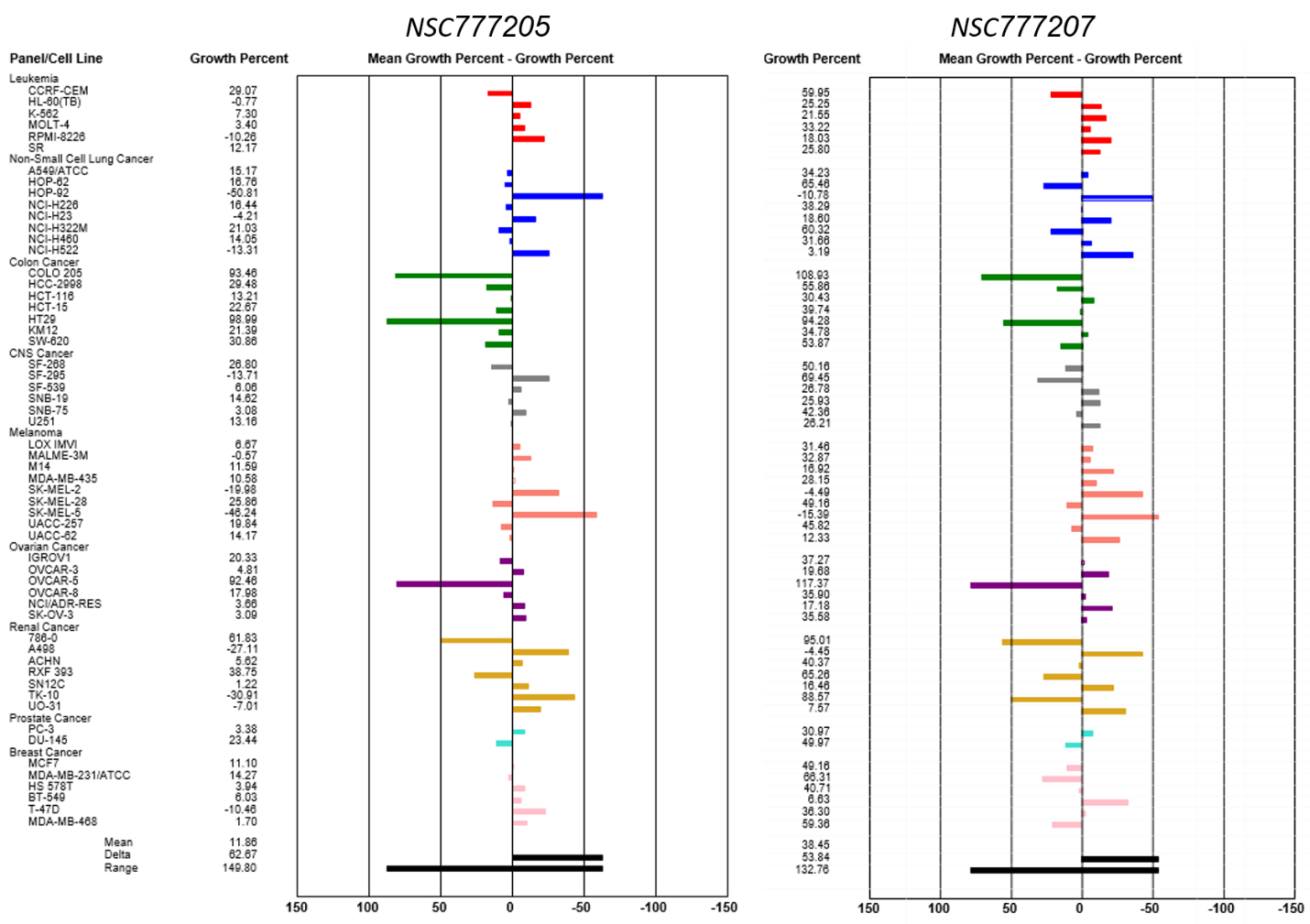


**Supplementary Figure 2:** Inhibitory activities of NSC777205 and NSC777207 against panels of 60 human cancer cell lines. Each cell line was treated with a single dose of 10 μM of NSC777205 and NSC777207. The zero point on the x-axis denotes the mean percentage of cell growth. The percentage growth of each cell line relative to the mean is represented by horizontal bars (y-axis). Growth inhibition was calculated relative to cells without drug treatment and the time-zero control. Extents of growth inhibition are indicated by values between 0 and 100, while lethality (cytotoxic effect) is indicated by values of < 0.


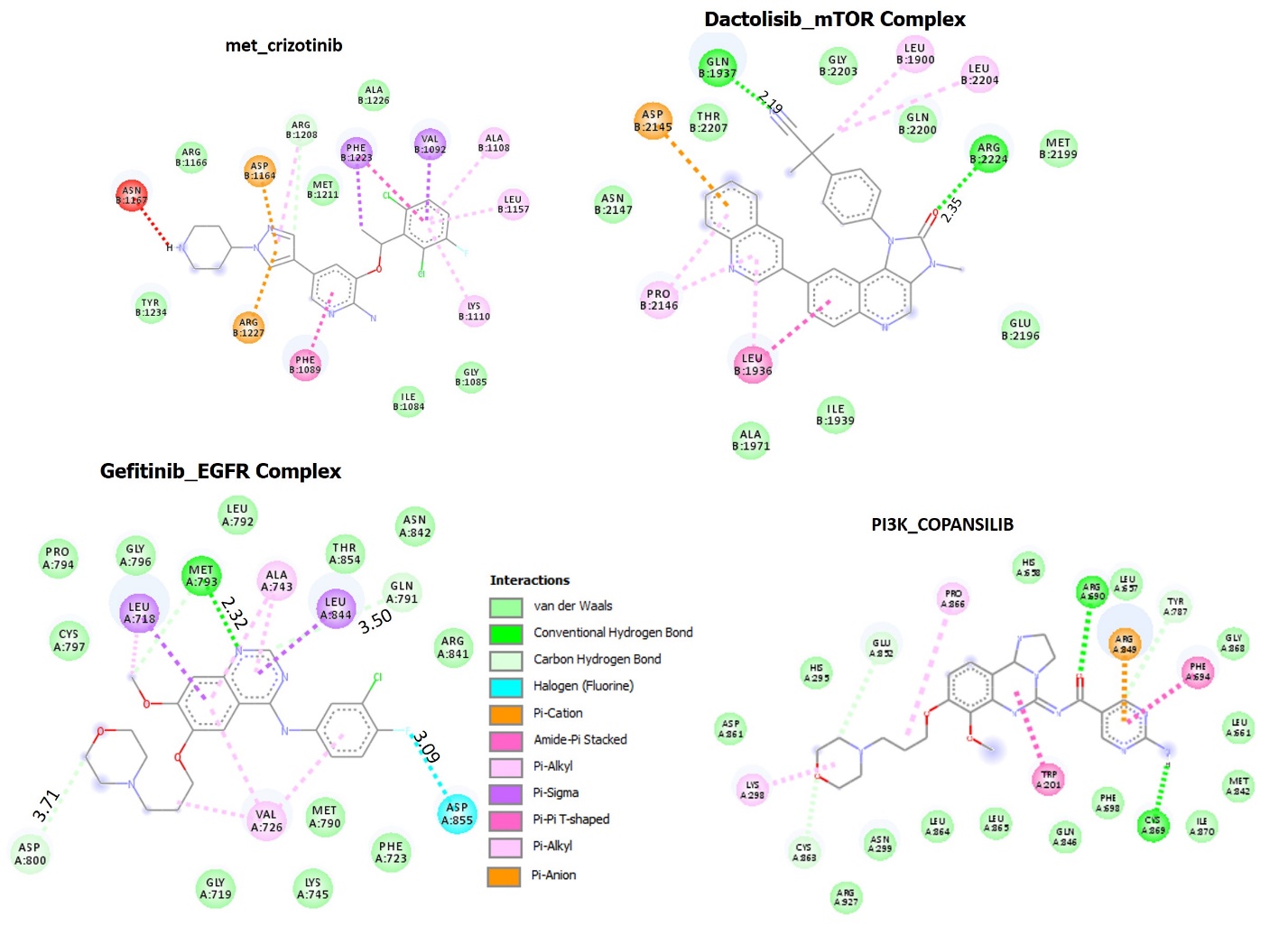


**Supplementary Figure 3:** The two dimensional (2D) representations of the docking profiles of PI3K (PDB:3APC), c-MET (PDB: 3RHK), EGFR (PDB: 5EDP), and mTOR (PDB: 5FLC) with their respective standard inhibitors
